# A non-canonical plant microRNA target site

**DOI:** 10.1093/nar/gku157

**Published:** 2014-02-21

**Authors:** Cécile Brousse, Qikun Liu, Linda Beauclair, Aurélie Deremetz, Michael J. Axtell, Nicolas Bouché

**Affiliations:** ^1^INRA, UMR1318, Institut Jean-Pierre Bourgin, RD10, F-78000 Versailles, France, ^2^AgroParisTech, Institut Jean-Pierre Bourgin, RD10, F-78000 Versailles, France and ^3^Department of Biology and Plant Biology Ph.D. Program, Pennsylvania State University, University Park, PA 16802, USA

## Abstract

Plant microRNAs (miRNAs) typically form near-perfect duplexes with their targets and mediate mRNA cleavage. Here, we describe an unconventional miRNA target of miR398 in *Arabidopsis*, an mRNA encoding the blue copper-binding protein (BCBP). *BCBP* mRNA carries an miR398 complementary site in its 5′-untranslated region (UTR) with a bulge of six nucleotides opposite to the 5′ region of the miRNA. Despite the disruption of a target site region thought to be especially critical for function, *BCBP* mRNAs are cleaved by ARGONAUTE1 between nucleotides 10th and 11th, opposite to the miRNA, like conventional plant target sites. Levels of *BCBP* mRNAs are inversely correlated to levels of miR398 in mutants lacking the miRNA, or transgenic plants overexpressing it. Introducing two mutations that disrupt the miRNA complementarity around the cleavage site renders the target cleavage-resistant. The *BCBP* site functions outside of the context of the *BCBP* mRNA and does not depend on 5′-UTR location. Reducing the bulge does not interfere with miR398-mediated regulation and completely removing it increases the efficiency of the slicing. Analysis of degradome data and target predictions revealed that the miR398-*BCBP* interaction seems to be rather unique. Nevertheless, our results imply that functional target sites with non-perfect pairings in the 5′ region of an ancient conserved miRNA exist in plants.

## INTRODUCTION

microRNAs (miRNAs) are essential small non-coding RNAs involved in multiple biological aspects, regulating gene expression post-transcriptionally. While miRNAs have the same importance in the development of both plants and animals, there are major differences between the two (1). The number of targets per miRNA is strikingly different: from a vast number of genes in mammalians, with reports describing as much as 60% of all mRNAs targeted (2), to a very limited amount in plants, with ∼150 mRNA/miRNA duplexes experimentally validated until now. Nevertheless, plant miRNAs target essential transcription factors and mutants impaired in their biogenesis are embryo-lethal or sterile (*dcl1, hen1* or *hyl1*). The mode of action is also different: plant miRNAs mostly direct the cleavage of their targets, while targeted mRNAs of animals are more prone to translational repression and/or destabilization. Moreover, most target sites in humans are found in 3′-untranslated regions (UTRs) that can sometimes contain multiple sites for a given miRNA, while plant target sites are often unique and located in open reading frames (ORFs). In animals, the complementarity between the ‘seed’ region (positions 2–8 corresponding to 5′ ends of miRNAs) and target RNAs was seen to be a crucial element for target specificity. But exceptions to this ‘seed rule’ are more common than previously thought. For instance, certain miRNA complementary sites perfectly match their miRNA, except for a single G-bulge opposite to the center of the seed region, as observed in 15% of argonaute (AGO)–miRNA interactions found in mouse brain (3). Centered sites exhibit 11–12 contiguous pairings to the central region of the miRNA and not the seed region (4). A recent study identifying miR155 targets by AGO immunoprecipitation in cells with diverse miR155 contents revealed that ∼40% of these targets contain mismatches in the seed region (5). In contrast, no plant targets with extensive mismatches have yet been functionally validated.

miR398 is a conserved plant miRNA targeting mRNAs encoding proteins that use copper as a cofactor, namely, the cytosolic copper/zinc superoxide dismutase1 (CSD1), the chloroplastic CSD2, a subunit of the mitochondrial cytochrome c oxidase (COX5b-1) and a copper chaperone for superoxide dismutase1 (CCS1) (6–9). The transcriptional dependency of *MIR398* genes to biotic and abiotic stresses is well-described (10). Copper itself plays a major role in the transcription of both *MIR398b* and *MIR398c*, two of the three *MIR* genes encoding mature miR398 in *Arabidopsis* (11,12), through action of the transcription factor SQUAMOSA promoter binding protein-like7 (SPL7) (13). Like all plant miRNAs, miR398 acts by guiding the AGO-mediated cleavage of targeted mRNAs, but, in addition, miR398 is likewise one of the few plant miRNAs that was described to trigger translational repression (6,14).

Here, we show that miR398 directs the cleavage of a target that was not previously identified. The 5′-UTR of the blue copper-binding protein (*BCBP*) mRNA contains a unique miR398 complementary site with a bulge of six nucleotides opposite to the miRNA 5′ region. *BCBP* mRNA levels are inversely correlated with miR398 levels, and changing two nucleotides in the miR398 site of *BCBP* renders the target cleavage-resistant in both *Arabidopsis* transgenic plants and *Nicotiana benthamiana* leaves. The *BCBP* site functions outside of the *BCBP* mRNA context in both 5′- and 3′-UTRs, and the bulge can be reduced without affecting miR398-mediated slicing. The natural position of the bulge, between nucleotides 6 and 7, is the only 5′ or central position of the target site compatible with AGO1 slicing. Finally, although the *BCBP* site seems to be rather unique in *Arabidopsis*, our bioinformatics analyses suggest that other similar duplexes might exist.

## MATERIALS AND METHODS

### Plant materials and growth conditions

*Arabidopsis thaliana* accession Col-0 is the reference wild-type (WT) used in this study. Surface-sterilized seeds were plated on a culture medium prepared as previously described (6). Culture conditions were as follows: 16/8 h day/night cycles, 100–150 µmol m^−^^2^ s^−^^1^ light intensity, 20/15°C day/night temperature and 65% humidity. The *spl7-1* mutant (SALK_093849) was described (13), and the *spl7-2* mutant corresponds to SALK_125385. The following *ago* alleles were used: *ago1-1* and *ago2-1* (15). *Nicotiana benthamiana* plants were grown at 22°C under 24-h light condition. One-month-old plants were used for infiltrations.

### Generation of constructs

To generate *Arabidopsis* transgenic plants, the genomic sequence of At5G20230 (*BCBP* gene), including 650 bp before the start codon, was PCR-amplified using the following two primers: At5G20230 attB1 F and At5G20230 attB2 R3 (Supplementary Table S5). The DNA fragment that corresponds to the *proBCBP*:*BCBP* construct was cloned into pDONR207 (Invitrogen) using a BP clonase. This construct was mutagenized to generate a clone carrying *proBCBP*:*2mBCBP*, using the QuikChange Site-Directed Mutagenesis kit (Stratagene) and the primers At5g20230 mut F2 and R2 (Supplementary Table S5). Constructs in pDONR207 were transferred to the gateway pGWB1 binary vector (16) using an LR clonase.

To generate the 5′-UTR fusions to the green fluorescent protein (GFP), the 5′-UTR of BCBP was first amplified using the oligos attB1 20230 5′-UTR F and attB2 20230 5′-UTR R (Supplementary Table S5) and cloned into pDONR207 with a BP clonase and then transferred to pGWB5 (16) between the *35S* and the *sGFP* sequences in transcriptional fusion with the GFP. *2mBCBP:GFP* was generated in the same way from the pDONR207 *proBCBP*:*2mBCBP* construct. Also, *4* and *2 nt* and ‘perfect’ bulge UTR fusions with the *GFP* were generated similarly, by successive mutageneses/clonings removing two nucleotides from the bulge each time. The *controlUTR*:*GFP* construct was generated using oligos specific of the AT5G66380 UTR (*ATFOLT1* gene), namely, attB1 AT5G66380 F and R (Supplementary Table S5). All clones were verified by sequencing.

Generation of dual-luciferase sensors has been described (17). Target site sequences with alignments are shown in Supplementary Table S4. Supplementary Table S5 contains oligonucleotide sequences.

### *Agrobacterium*-mediated transient expression in *N. benthamiana*

Constructs carrying fusions between the different UTRs and the reporter genes were transferred to *N. benthamiana* leaves as described (18), with agrobacteria resuspended at OD 0.05 for GFP experiments and 0.25 for F-Luc. The pBARN*-35S*:*MIR398c* construct (11) was transferred with agrobacteria resuspended at OD 0.75 for GFP experiments and 0.25 for F-Luc. The empty pBARN-*35S* that replaced the pBARN-*35S*:*MIR398c* construct when no miR398 was required (Figures 4A and B and 5A; *−miR398*) was transferred to plants with agrobacteria resuspended at OD 0.75. Leaves were infiltrated and after 48 h, total proteins and RNAs were extracted to determine the corresponding GFP or luciferase levels.

### RNA analysis

Total RNA was extracted as described previously (19), and small RNAs were probed with miR398 as described previously (6). miR398 ^32^P end-labeled oligonucleotide probe was 5′-CAGGGGTGACCTGAGAACACA-3'. For mRNA expression analyses by quantitative reverse transcriptase polymerase chain reaction (qRT-PCR), total RNAs were extracted in bulks of 10–15 *Arabidopsis* plants grown for 15 days *in vitro*, with or without copper added to the medium. RNAs were treated as previously described (6), and we used forward and reverse primers (spanning the miR398 complementary site) specific of *CCS1*, *CSD1*, *CSD2* and *BCBP* (Supplementary Table S5). To identify the cleavage sites by 5′-rapid amplification of cDNA ends (RACE) PCR, we used the *GeneRacer* kit (Invitrogen), as described previously (20), with oligos specific of At5G20230 (*BCBP* gene) in *Arabidopsis* and GFP in *N. benthamiana* infiltrations (Supplementary Table S5). For northern blot and qRT-PCR analyses in *N. benthamiana*, high-molecular-weight total RNA fractions were extracted using the *RNeasy* kit (Qiagen). For northern blotting, 3 µg of RNA was loaded on a denaturing gel and transferred to a membrane. The *GFP* mRNA was probed with a 170-bp *GFP* DNA fragment (localized in the middle of the *GFP* gene) amplified using the oligos GFPr/GFPf (Supplementary Table S5) and radiolabeled using the *Prime-a-gene* labeling system (Promega). For qRT-PCR, cDNAs were obtained with the QuantiTect Reverse Transcription Kit (Qiagen), using 1 µg of total RNA. qRT-PCR, using oligos flanking the target site in the case of *F-Luc*, was performed using QuantiTect SYBR Green RT-PCR Kit (Qiagen) to derive *F-Luc* to *R-Luc* ratios.

### Dual-luciferase assay

Dual-luciferase assays were performed as previously described (17).

### Western blotting

Antibodies against GFP were obtained from Roche Applied Science (ref: 11814460001) and used according to the manufacturer’s instructions. Western blotting was performed as described previously (6) with 30 µg of total proteins loaded per sample.

### Bioinformatics

The minimum free energy (MFE) of duplexes was calculated with the *RNA-hybrid* program (21,22). Nine *Arabidopsis* degradome libraries (Supplementary Table S2) were analyzed separately by CleaveLand4, using the TAIR10 mRNAs, and all *Arabidopsis thaliana* miRBase entries from miRBase 20. CleaveLand4 and GSTAr are freely available at http://axtell-lab-psu.weebly.com/cleaveland.html.

## RESULTS

### Isolation of a new type of miRNA target in *Arabidopsis*

**Figure 1.**
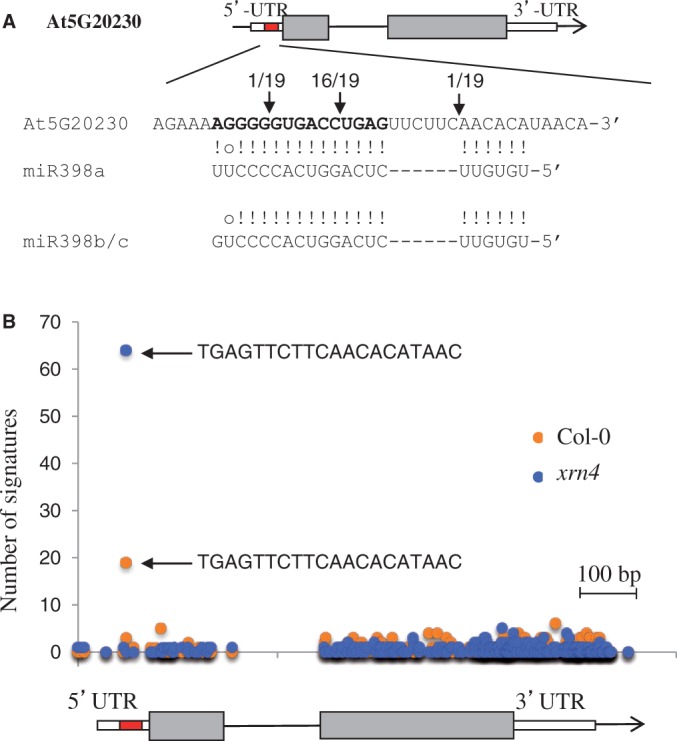
*BCBP* contains an unconventional miR398 target site. (**A**) Schematic presentation (drawn to scale) of the *BCBP* gene (*At5G20230*). The location of the miR398 target site is shown (red box), exons are represented by gray boxes and UTRs by white boxes. The sequence (*Arabidopsis* accession Col-0) of the miR398 complementary site (in bold) in the *BCBP* mRNA is aligned with sequences of both miR398a and miR398b/c. Cleavage was experimentally validated by a modified version of the 5′-RACE PCR (20). The arrow indicates the 5′ terminus cleavage product for the number of clones mentioned. (B) Degradome data (24) corresponding to the signatures matching *At5G20230* in both Col-0 WT plants and *xrn4* mutants. The arrows indicate the signatures that correspond to the miR398 target site identified in (A). The gene model is presented below.

We previously demonstrated that the CCS1 is targeted by miR398 (6). *CCS1* mRNAs escaped all previous bioinformatics predictions because the duplex formed with miR398 exceeded the number of mismatches allowed by the algorithms of prediction. By changing these parameters, applying more flexible rules to both pairings in the 5′ region of the miRNA and GU contents (i.e. a score of 3.5, allowing a maximum of six G:U pairs, one indel and five mismatches), we retrieved eight new putative miR398 targets (Supplementary Table S1). A cleavage product was identified, by 5′-RACE PCR (20), only in the predicted target site for At3g27200, encoding a plastocyanin-like protein (Supplementary Figure S1), which is a member of a large family of copper-binding proteins (23). We extended our analysis to all members of the family by aligning mRNA sequences with miR398, and we found no predicted stable duplexes for most of the gene family members, except for one gene, At5g20230, encoding a BCBP. The putative miR398 site is localized in the 5′-UTR of the gene; however, there was a bulge of six nucleotides in the 5′ region of the miRNA/target duplex, opposite to the miRNA (Figure 1A and Supplementary Figure S2), which has not been observed for validated plant miRNA targets. Surprisingly, we found, by 5′-RACE PCR, in two biological replicates, that *BCBP* mRNAs were cleaved at the miR398 predicted target site, opposite to nucleotides 10 and 11 of the miRNA guide (Figure 1A), a position diagnostic of AGO1 slicing. This was confirmed by publicly available degradome data (Figure 1B) from both Col-0 plants and *xrn4* mutants (24). Notably, the degradome evidence was stronger in the *xrn4* data, consistent with the fact that *xrn4* mutants have increased accumulation of the 3′ products of miRNA-mediated slicing (25). Finally, in *ago1-1* mutants, we could not amplify the 3′ fragment resulting from the cleavage of *BCBP* mRNAs, while we detected the uncleaved *BCBP* transcript (Supplementary Figure S3). We observed the inverse situation for both Col-0 and *ago2-1*, where the cleavage product was clearly detected in contrast to uncleaved *BCBP* transcripts (Supplementary Figure S3). This indicates that AGO1, the major contributor to miRNA-mediated cleavage in *Arabidopsis* (15), is likely to slice *BCBP* mRNAs.

### Levels of *BCBP* mRNAs and miR398 are inversely correlated

**Figure 2.**
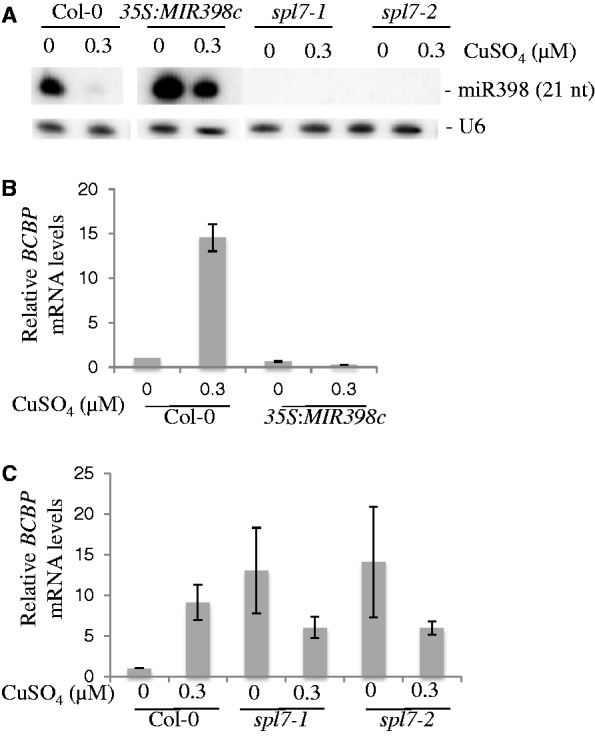
*BCBP* mRNA levels are inversely correlated with miR398 levels. (**A**) RNA gel blots (1 µg total RNAs) prepared from 12-day-old seedlings carrying a *35S::MIR398c* construct, two *spl7* alleles (Supplementary Figure S5) and the corresponding WT (Col-0) plants grown with (0.3 µM CuSO_4_) or without copper, as indicated. miR398 was detected with a radiolabeled oligonucleotide probe. *U6* RNA was used as a loading control. (**B**) qRT-PCR quantification of *BCBP* mRNAs in WT (Col-0) and *35S:MIR398c* plants described in (A). Average values of three technical qPCR repeats were compared with the level of mRNA in WT plants grown without copper, arbitrarily fixed to 1. Error bars indicate SD (*n* = 6). Results for known miR398 mRNA targets (*CSD1*, *CSD2* and *CCS1*) using the same RNA samples are presented in Supplementary Figure S4. (**C**) qRT-PCR quantification of *BCBP* mRNAs in WT (Col-0) and *spl7* mutants plants described in (A). Average values of three technical qPCR repeats were compared with the level of mRNA in WT plants grown without copper, arbitrarily fixed to 1. Error bars indicate SD (*n* = 6). Results for known miR398 mRNA targets (*CSD1*, *CSD2* and *CCS1*) using the same RNA samples are presented in Supplementary Figure S4.

To determine whether *BCBP* mRNA levels respond to miR398, we generated plants overexpressing the precursor of *MIR398c* (11). We grew these plants and the corresponding WT Col-0 on plates supplemented with or without copper because miR398 levels are dependent on copper availability (11,12). miR398 accumulation, determined by northern blot, decreases in Col-0 plants when copper is added to the medium but remains at high levels independent of copper concentrations in transgenic plants harboring a *35S*::*MIR398c* construct (Figure 2A). We determined the mRNA levels of three previously identified miR398 targets, *CSD1*, *CSD2* and *CCS1* (10), as well as *BCBP* mRNA levels. The accumulation of all four mRNAs, including *BCBP*, was inversely linked to the quantities of miR398, in both transgenic and Col-0 plants (Figure 2B and Supplementary Figure S4A). Indeed, all mRNAs were detected only in the absence of miR398.

The SPL7 transcription factor activates the transcription of several genes involved in copper homeostasis, including *MIR398b* and *MIR398c*, the main contributors to the production of miR398 in *Arabidopsis* (26). Thus, in *spl7* mutants (Supplementary Figure S5), the global levels of miR398 are remarkably reduced compared with those in Col-0 plants, independent of the copper concentrations in the medium (Figure 2A). Levels of *CSD1*, *CSD2*, *CCS1* and *BCBP* mRNAs were inversely correlated to miR398 levels in two *spl7* mutant alleles (Figure 2C and Supplementary Figure S4B). We concluded from these experiments that *BCBP* transcript levels are tightly linked to the quantities of miR398 in Col-0, transgenic plants overproducing miR398 and mutants lacking the miRNA. However, this could be the consequence of an indirect effect of the miRNA; thus, we decided to manipulate the miR398 complementary site of *BCBP*.

### miR398-mediated repression of *BCBP* depends on central base pairs

**Figure 3.**
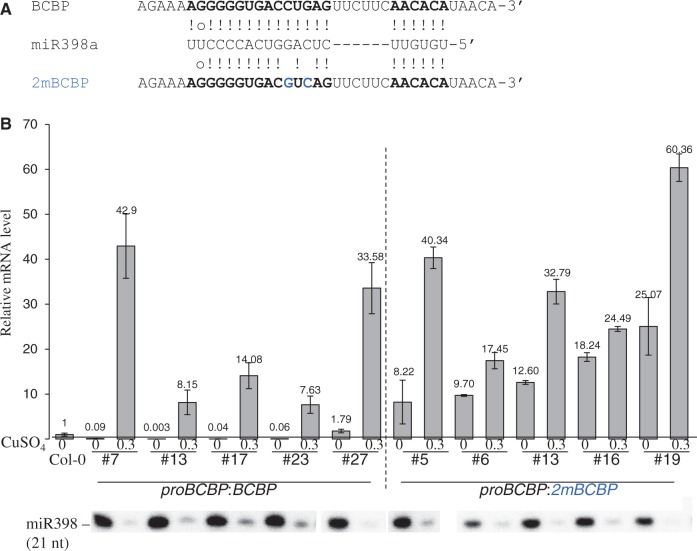
Central pairing is required for miR398-mediated repression of *BCBP.* (**A**) Pairing of miR398a with the corresponding complementary sites of both *BCBP* and an miR398-resistant form of *BCBP* (*2mBCBP*) containing two nucleotides (in blue) that disrupt the complementarity. (**B**) qRT-PCR quantification of *BCBP* mRNAs in transgenic plants (T3 generation) carrying either a *BCBP*-unmodified cDNA or a *2mBCBP* version, driven by its own promoter (*proBCBP*). Plants were grown for 12 days *in vitro*, with (CuSO4 0.3 µM) or without copper added to the medium. *MIR398b/c* genes are transcribed in the absence of copper. Total RNAs were extracted in bulk (10–15 plants) to prepare the corresponding cDNAs and perform the qRT-PCR. Results are presented for five independent transgenic lines carrying *proBCBP*:*BCBP* (lines #7, #13, #17, #23 and #27) and five independent lines for *proBCBP*:*2mBCBP* (lines #5, #6, #13, #16 and #19). Error bars indicate SD (*n* = 5). One microgram of total RNAs was analyzed by northern blot to control miR398 levels.

*Arabidopsis* transgenic plants expressing the *BCBP* cDNA driven by the *BCPB* native promoter were generated. Compared with the WT control (*proBCBP*:*BCBP*), the *proBCBP*:*2mBCBP* transgene contained two silent mutations disrupting the complementarity between miR398 and *BCBP* around the cleavage site (Figure 3A). Transformed plants were selected and after two generations, plants containing at least one homozygous transgene were grown with or without copper. *BCBP* mRNA levels were determined by qRT-PCR analyses. In five independent *proBCBP*:*2mBCBP* lines, *BCBP* mRNAs were highly expressed even when miR398 was plentiful. In contrast, *proBCBP*:*BCBP* control lines were sensitive to miR398 induction (Figure 3B). Therefore, changing two nucleotides in the miR398 complementary site of *BCBP* 5′-UTR is sufficient to modify the miRNA-based regulation. We concluded from this experiment and the 5′-RACE PCR mapping that miR398 mediates the cleavage of *BCBP* mRNA, despite the unconventional duplex formed between the miRNA and the target.

### The *BCBP* site functions outside of the *BCBP* mRNA context

**Figure 4.**
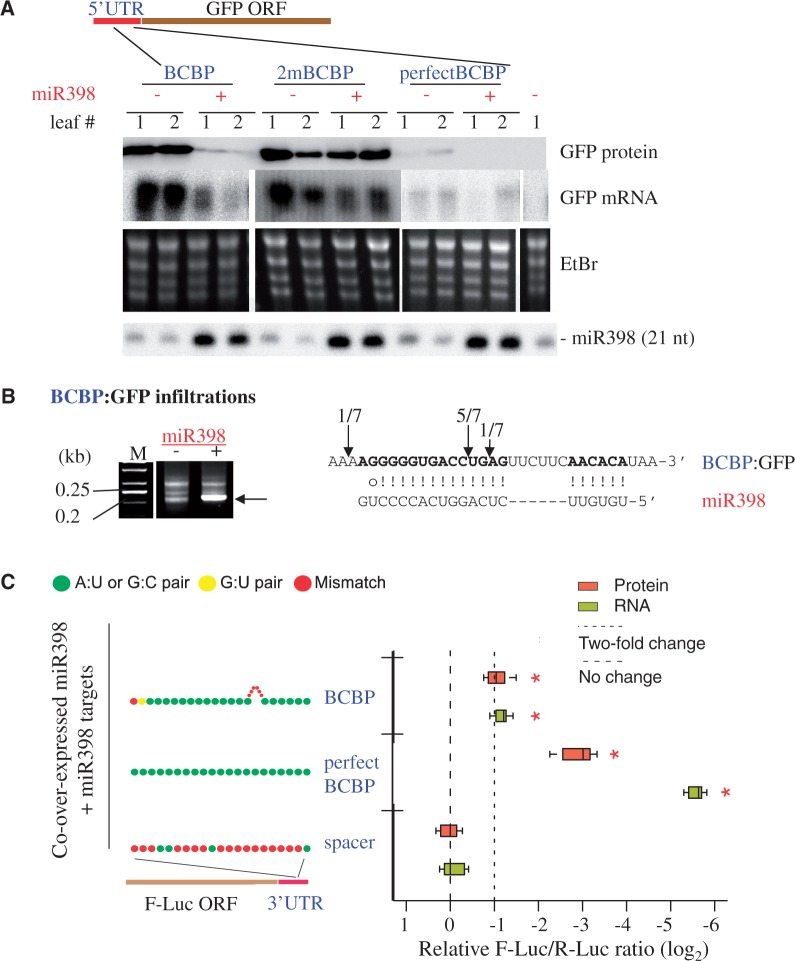
The *BCBP* site functions outside of the *BCBP* mRNA context in both 5′- and 3′-UTRs. (**A**) *BCBP* 5′-UTR fusion with the *GFP* gene. Western and northern blot analyses to determine the levels of the GFP protein and mRNA in two independent *N. benthamiana* leaves (notated ‘1’ and ‘2’) infiltrated with the constructs indicated. The *BCBP*:*GFP* construct corresponds to the 5′-UTR of *BCBP* fused to the *GFP*. *2mBCBP* has two mismatches around the cleavage site that disrupt the complementarity (Figure 3A). *perfectBCBP* is perfectly complementary to miR398. miR398 was detected with a radiolabeled oligonucleotide probe. (**B**) 5′-RACE PCR analyses of the *BCBP* site fused to the 5′-UTR of *GFP*. Total RNAs from *N. benthamiana* leaves infiltrated with the *BCBP*:*GFP* construct were analyzed by 5′-RACE PCR. The arrows indicate the DNA fragments cloned and sequenced to map the cleavage sites (on the right) located within the 5′-UTR. M; *GeneRuler 50-bp DNA ladder* (Fermentas). (**C**) *BCBP* 3′-UTR fusion with the *F-Luc* gene. Boxplots summarize results from nine independent replicates and show the median (thick line), extent of the 1st to 3rd quartile range (box), values extending to 1.5 times the interquartile range (whiskers), and outliers (black circles). Asterisks indicate significant differences comparing with the spacer control (*P* < 0.05, analysis of variance–Tukey’s honest significance difference). Colored dots show the schematic base-pairing pattern between miR398 and its targets with the target 5′ ends on the left.

To test whether the *BCBP* target site can function outside of its native context, we fused the corresponding *Arabidopsis BCBP* 5′-UTR sequence to the *GFP* and placed the fusion behind a constitutive *35S* promoter. This *35S*:*BCBP_5*′*-UTR*:*GFP* construct was co-expressed with a *35S*::*MIR398c* construct in transient-expression assays in *N. benthamiana* leaves. Levels of both mRNAs and GFP protein were drastically reduced when leaves were co-infiltrated with the two constructs (Figure 4A; *BCBP +miR398*), but not when an empty vector replaced the miR398 construct as a control (Figure 4A; *BCBP −miR398*). We monitored, by 5′-RACE PCR, slicing of the *BCBP_5*′*-UTR:GFP* mRNAs, and we found a cleavage product only when the precursor of miR398c was co-expressed (Figure 4B). We concluded that miR398 can trigger the subsequent degradation of a chimeric *GFP* mRNA containing the *BCBP* 5′-UTR. In a similar experiment, we co-infiltrated a GFP miR398-resistant form (containing central mismatches) with or without miR398, and we observed that the levels of both GFP mRNAs and protein were detected similarly in all conditions (Figure 4A; *2mBCBP +* and *−miR398*). As observed in *Arabidopsis* transgenic plants (Figure 3), modifying the complementarity site of *BCBP* abolishes the miRNA-based post-transcriptional regulation. As control, we fused the GFP to a UTR devoid of the miR398 site (corresponding to *At5G66380*) (Supplementary Figure S6; *controlUTR*).

It has been suggested that target sites in the 5′-end of a target and/or in the 5′-UTR are stronger (27), more tolerant of central mismatches and more prone to translational repression (28). We used a recently developed dual-luciferase transient expression system in *N. benthamiana* (17) to test whether the *BCBP* target site could function outside of the 5′-UTR context. A *BCBP* site was engineered into the 3′-UTR of a firefly luciferase gene (*F-Luc*) that was co-expressed with an miR398 overexpressor. Relative accumulation of the F-Luc sensor was evaluated at both the mRNA (via qRT-PCR with primers spanning the target site) and protein (by quantification of luciferase activities) levels using *Renilla* luciferase (*R-Luc*), expressed from the same T-DNA, as an internal control. Levels of both F-Luc mRNA and protein were reduced when the *BCBP* target site was placed in the 3′-UTR (Figure 4C; *BCBP*), indicating that the target site functions in both 5′- and 3′-UTRs. As a positive control, we used a *BCBP* site with no bulge (Figure 4C; *perfect BCBP*), and as a negative control, we used a 21-nt spacer of random sequence (Figure 4C; *spacer*). Altogether, our results indicate that the *BCBP* site, placed in either 3′- or 5′-UTRs, triggers the cleavage and repression of reporter genes in transient assays.

We next compared the efficiency of repression of the bulged *BCBP* site with that of a perfectly matched site. Surprisingly, when a perfectly matched construct was co-infiltrated with an empty vector, we detected very low levels of GFP mRNA and protein (Figure 4A; *perfect BCBP −miR398*). We hypothesize that the endogenous *N. benthamiana* miR398 could efficiently mediate the degradation of this chimeric construct. Indeed, adding miR398 resulted in an even more efficient processing of the perfect site sensor (Figure 4A; *perfect BCBP +miR398*) because we detected no GFP protein at all. Additionally, we confirmed these results using the dual-luciferase system, where a perfect site conferred much stronger repression than the bulged *BCBP* site (Figure 4C).

### Size and location of the bulge in the *BCBP* target site affect repression efficiency

**Figure 5.**
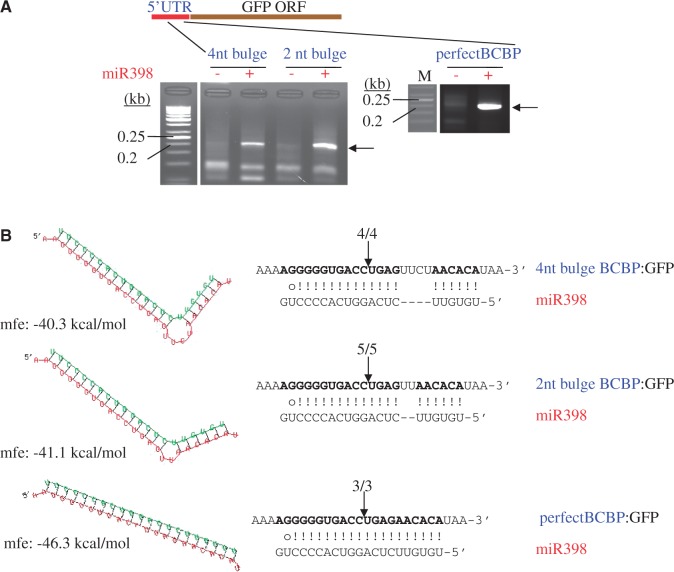
Smaller bulges are also functional. (**A**) 5′-RACE PCR analyses of the *BCBP* site fused to the 5′-UTR of *GFP*. Total RNAs from *N. benthamiana* leaves infiltrated with the *BCBP*:*GFP* construct indicated were analyzed by 5′-RACE PCR. The arrows indicate the DNA fragments cloned and sequenced to map the cleavage sites (**B**) located within the 5′-UTR. M; *GeneRuler 50-bp DNA ladder* (Fermentas).

To understand the impact of the large bulge on slicing, we first reduced it by stepwise removal of two nucleotides each time and placed these modified target sites in 5′-UTR of the *GFP* reporter. We co-infiltrated these constructs in *N. benthamiana* leaves with or without the *35S*::*MIR398c* construct. For all constructs, we detected, by 5′-RACE PCR, a fragment that corresponded to the cleaved *GFP* mRNA only when miR398 was co-expressed (Figure 5A). We also mapped the cleavage site to the expected location between the 10th and 11th nucleotides (Figure 5B). This indicates that miR398 cleaves the target site regardless of the presence or size of the bulge.

**Figure 6.**
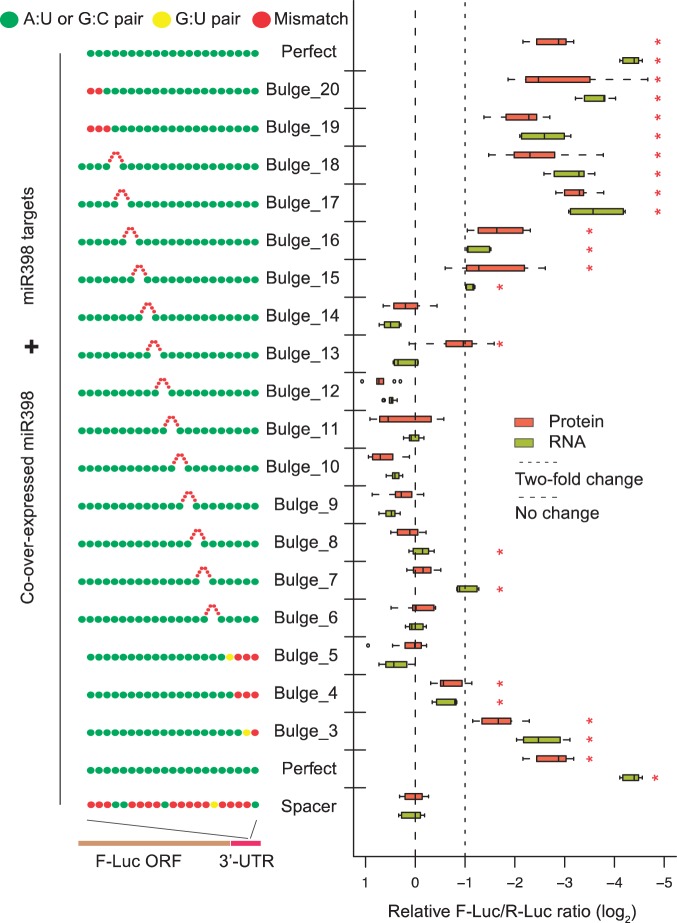
Effects of 6-nucleotide bulges present at different positions. Engineered miR398 target sites in 3′-UTR F-Luc sensors. Transient dual-luciferase assays in *N. benthamiana* leaves with display conventions as in Figure 4C. Red dots on target 5′ and 3′ ends represent dangling ends as predicted by RNAcofold. miRNA-target site alignments are presented in Supplementary Table S4.

Next, we examined the tolerance for six-nucleotide bulges at different positions of the miRNA/target duplex using the dual-luciferase 3′-UTR sensor system (17). Large bulges at the ends of the duplex are unlikely to form (based on thermodynamic predictions), so the Bulges 3–5 and Bulges 19–20 sensors most simply had unpaired residues at the ends (Figure 6). Bulges in the 5′ and central regions of the target fully abolish regulation, with the exception of Bulge 7, where the bulge is between positions 6 and 7, precisely as in the *Arabidopsis BCBP* site (Figure 6). In contrast to the *Arabidopsis BCBP* site, which has 3′-end mismatches, the 3′ ends of our bulge-scanning sensors were all perfectly paired. We noted that the target site seems to be more efficiently processed when the 3′ mismatches are present (compare Figure 4C *BCBP* with the Bulge 7 sensor Figure 6), suggesting that the 3′-mismatches enhance function of the bulged *BCBP* site. Finally, bulges are tolerated in the 3′ end of the site, as Bulges 15–18 are functional. This is consistent with the fact that, in general, plant miRNA target sites are more tolerant of disruptions in the 3′ end.

Thus, reducing the bulge at its natural position (i.e. between positions 6 and 7) does not interfere with miR398-mediated regulation, completely removing it increases the efficiency of repression and position 6–7 seems to be the only 5′ or central position of the target site that tolerates a large bulge.

### The *BCBP* target site is rather unique

We extended our analysis to other miRNAs in *Arabidopsis* to identify putative miRNA/target duplexes carrying large target-side bulges. Traditional plant miRNA-target aligners fail to find sites with large bulges. Therefore, we developed the general small RNA-transcriptome aligner (GSTAr), which uses RNAplex (29) as an alignment engine and captures alignments based on MFE calculations instead of simple mismatch and gap penalties. Comparing miRBase 20 *Arabidopsis* miRNAs against the TAIR10 cDNA models, we identified 7794 alignments with one or more target-side bulges of four nucleotides or larger. We next applied an MFE ratio cutoff of 80% (i.e. the MFE of the alignment over the MFE of a perfectly complementary alignment was ≥0.8). We found 77 alignments with MFE ratios ≥80%, including the miR398/*BCBP* site with a value of 84%. We tried to detect evidence of slicing among these duplexes by analyzing nine *Arabidopsis* degradome libraries (Supplementary Table S2) with CleaveLand4, an updated version of the CleaveLand pipeline for degradome analysis (30). Of the 77 alignments, 67 had no data at all, whereas the remaining ones showed little evidence for slicing (Supplementary Figure S7). The duplex formed between *BCBP* and miR398 was the only exception: it was identified in various degradome libraries, and for some of them, it was associated with a high number of hits corresponding to the cleavage site. Therefore, the miR398-mediated slicing of *BCBP* appears to be somewhat unique among potential miRNA/target duplexes with large bulges.

## DISCUSSION

miR398 is a conserved and ancient plant miRNA that targets mRNAs encoding proteins using copper as a co-factor, including the two superoxide dismutases CSD1 and CSD2 and the chaperone protein CCS1 delivering copper to both of them. miR398 itself is controlled by a copper-responding transcription factor, SPL7. Here, we identified two new mRNAs encoding copper-containing proteins of unknown function, controlled by miR398, one is a plastocyanin-like protein (At3G27200; Supplementary Figure S1) and the other one is BCBP. *At3G27200* carries an miRNA site similar to others identified in plants, with a high level of complementarity along the whole sequence of the site. *BCBP*, however, is an exception. Indeed, the miRNA and its target form an unconventional duplex with a bulge of six nucleotides in between miRNA positions 6 and 7.

Despite this very specific pairing, the *BCBP* target site shares various characteristics with other plant miRNA targets. First, we found a strong inverse correlation between the levels of miR398 and the *BCBP* mRNA in all conditions tested: WT plants with levels of miR398 artificially controlled, mutants lacking the miRNA and transgenic plants that overexpress miR398 (Figure 2). Although the precise function of BCBP remains unknown, we noticed that *BCBP* mRNAs seem to be at their highest level of detection in pollen (Supplementary Figure S8) and that BCBP protein is also highly detected in proteome analyses of mature pollen (31). miR398 was one of the few conserved miRNAs noticeably absent from small RNA sequences obtained from pollen of rice (32). These data suggest an inverse correlation between miR398 and BCBP levels occurs in a tissue-specific manner and possibly to a more specific function of BCBP in pollen. Second, we confirmed slicing of *BCBP* at the canonical position (between nucleotides 10–11) by both 5′-RACE PCR analyses (Figure 1A) and from degradome data (Figure 1B and Supplementary Figure S7). Cleavage observed at this position is indeed diagnostic of slicing by AGO in many species, including *Drosophila* (33), yeast (34) and plants (20). We modified the complementary site by replacing two nucleotides around the cleavage site, and we rendered BCBP insensitive to miR398 levels in both transgenic *Arabidopsis* plants stably transformed (Figure 3) and transient assays in *N. benthamiana* leaves (Figure 4A). In plants, disrupting the complementarity between miRNA and its target around position 10/11 usually has drastic consequences on miRNA control, for instance by modifying the target site of miR164 in CUP-SHAPED COTYLEDON2 (35), or the miR398 target site in *CCS1* (6). Third, the MFE of the duplex formed between miR398 and *BCBP* is predicted to be low (−39.5 kcal/mol), and consequently, the miRNA/target hybrid is likely very stable. This value is within the range of others obtained for different miRNA/target couples that were experimentally validated in plants (Supplementary Table S3). In animals, certain non-canonical complementary sites compensate for the lack of complementarity in the 5′ end by increased pairing in the 3′ end (5), emphasizing the importance of overall duplex stability in both plant and animals. Still, perfect miR398 sites are more stable (Figure 5B) and are repressed much more efficiently than the *BCBP*-bulged target site (Figure 4A and C). Whether the *BCBP* target site was selected to be processed sub-optimally, for instance to maintain a certain threshold of mRNA despite the miRNA-control, remains to be determined.

Plant miRNAs were once thought to exert their control mainly by mediating target cleavage, in contrast to animals, in whom miRNAs act by repressing translation. Only few plant miRNAs were initially shown to act by both cleavage and translational control, miR398 being one of them (6,14). A recent report provided new insight into translational repression in plants (28), using a slicing-deficient mutant of *Arabidopsis* AGO1 that can only repress translation. Translation is effectively repressed in an *in vitro* system when sites fully complementary to the miRNA are positioned in both UTRs or ORFs. But, interestingly, non-cleavable sites with central mismatches of up to four nucleotides were only capable of translational repression when positioned in 5′-UTRs. In animals, the vast majority of sites are located in 3′-UTRs (36), but in plants, most miRNAs target ORFs. miR398 is an exception because most of its targets contain sites in their 5′-UTRs, including *CCS1*, *CSD1* and, now, *BCBP*. Determining the precise pairing rules that trigger translational repression in plants will help to understand if miR398 target sites are more prone to translational control because of their singular 5′-UTR location.

Most of the known targets for plant miRNAs were discovered using computational predictions based on high levels of homologies between the miRNA and the target, followed by experimental validations (5′-RACE PCR or degradome analyses) designed to identify the cleavage site. In this study, we clearly demonstrated that miRNA/target duplexes existing in the wild can escape one of the main rule used for computational predictions in plants: the region that corresponds to the 5′ part of the miRNA is not necessarily strictly homologous to the target site. The list of plant miRNA targets is probably still expandable. We noticed that the natural position of the bulge in BCBP is the only one compatible with large bulges (Figure 6). This position is also the one that shows the highest percentage of mismatches within the 5′ region of the miRNA, of 139 duplexes experimentally validated in *Arabidopsis* (Supplementary Figure S9). By aligning the sequences of *BCBP* miR398 sites from different *Arabidopsis* accessions, we found two polymorphic sites, one located within the bulge and the other in the 3′ region of the miRNA/target duplex (Supplementary Figure S10A). Moreover, the sequences of *BCBP*-like genes from other plant species (Supplementary Figure S10B) indicate that in the wild, the size and sequence of the bulge are variable, but not the sequences homologous to the miRNA. miRNAs of animals are only complementary to their targets in limited regions of the miRNA/target duplex. Although it is unlikely that such targets would be cleaved in plants, the fact that translational repression remains very efficient when bulges of up to four nucleotides are introduced around the cleavage site (28) may open a new way toward the discovery of plant targets that are only controlled by translational repression. In support of this hypothesis, we show that other target sites with bulges might exist in *Arabidopsis*, and it is tempting to speculate that they are regulated through translational repression because no evidence of slicing was detected (Supplementary Figure S7). If this proves true, *BCBP* might be a unique site with translation repression characteristics that has remained cleavable.

## SUPPLEMENTARY DATA

Supplementary Data are available at NAR Online.

## FUNDING

Agence Nationale de la Recherche [Project 11-JSV7-0013 to N.B.] and the US National Science Foundation [Award 1121438 to M.J.A.]. Funding for open access charge: Agence Nationale de la Recherche [Project 11-JSV7-0013].

*Conflict of interest statement*. None declared.

## Supplementary Material

Supplementary Data
