# Supplementary material for: A non-canonical plant microRNA target site
Source: Nucleic Acids Res. 2014 Feb 21;42(8):5270–9. doi: 10.1093/nar/gku157 (PMC4005643; doi:10.1093/nar/gku157)
Supplement: Supplementary Data [file supp_42_8_5270__index.html]

A non-canonical plant microRNA target site — A non-canonical plant microRNA target site — Supplementary Data 

# A non-canonical plant microRNA target site

## Supplementary Data

files

**Files in this Data Supplement:**

- Supplementary Data - pdf file
