# Supplementary material for: A non-canonical plant microRNA target site
Source: Nucleic Acids Res. 2014 Feb 21;42(8):5270–9. doi: 10.1093/nar/gku157 (PMC4005643; doi:10.1093/nar/gku157)
Supplement: Supplementary Data [file supp_gku157_nar-00091-a-2014-File003.pdf]

SUPPLEMENTAL FIGURE LEGENDS

Figure S1. *At3G27200* is targeted by miR398

(A) Schematic presentation (drawn to scale) of *At3G27200*. The location of the miR398 target site is shown (black box), exons are represented by grey boxes and UTRs by white boxes. The sequence (Arabidopsis Col-0) of the miR398 recognition site (in bold) in the *At3G27200* mRNA is aligned with sequences of both miR398a and miR398b/c. Cleavage was experimentally validated by a modified version of the 5'-RACE PCR (20). The arrow indicates the 5' terminus cleavage product for the number of clones mentioned.

(B) Duplex formed between miR398a and *At3G27200*. The arrow indicates the cleavage site. The Minimum Free Energy (MFE) of the duplex (calculated with the *RNA-hybrid* program (21,22) is indicated.

(C) Degradome data (24) corresponding to the signatures matching *At3G27200* in both Col WT plants and *xrn4* mutants. The arrows indicate the signatures that correspond to the miR398 target site identified in (A). The gene model is presented below.

Figure S2. Duplex formed between miR398a and *BCBP* 5'-UTR mRNA

The arrow indicates the cleavage site determined by 5'-RACE PCR (Figure 1). The *mfe* of the duplex is indicated. *mfe* of a perfect match hybrid for miR398a with itself is 45.7 kcal/mol and 46.9 kcal/mol for miR398b/c.

Figure S3. *BCBP* mRNA cleavage is mediated by AGO1

5'-RACE PCR analyses in *ago1-1*, *ago2-1* and Col-0 plants. Total RNAs were extracted from bulks of plants cultivated *in vitro* for 15 days (*ago1-1* plants were selected based on their phenotype and grown for an extra week). Oligos (Table S5) specific to *BCBP* (A) or *CCS1* (B) mRNAs were used for the 5'-RACE PCR. *BCBP* uncleaved mRNA (C) was amplified with oligos (Table S5) localised around the miR398 cleavage site. M; *GeneRuler 50 bp DNA ladder* (Fermentas).

Figure S4. *CSD1*, *CSD2* and *CCS1* mRNA levels are inversely correlated with miR398 levels

(A) qRT-PCR quantification of known miR398 mRNA targets (*CSD1*, *CSD2* and *CCS1*) in WT (Col-0) and *35S:MIR398c* plants described in Figure 2A. Average values of three technical qPCR repeats were compared to the level of mRNA in WT plants grown without copper, arbitrarily fixed to 1. Error bars indicate SD (n=6).

(B) qRT-PCR quantification of known miR398 mRNA targets (*CSD1*, *CSD2* and *CCS1*) in WT (Col-0) and *spl7* mutants (Figure S5) described in Figure 2A. Average values of three technical qPCR repeats were compared to the level of mRNA in WT plants grown without copper, arbitrarily fixed to 1. Error bars indicate SD (n=6).

Figure S5. Phenotype of the *spl7* mutants

(A) Schematic presentation (drawn to scale) of the *SPL7* gene (*At5G18830*). The position of the T-DNA inserts in *spl7-1* (SALK_093849) or *spl7-2* (SALK_125385) knockout mutants is indicated. *spl7-1* has been described previously (13).

(B) Phenotype of *spl7* mutants grown for two months under standard greenhouse conditions.

Figure S6. Controls for the GFP/miR398 infiltrations

The 35S:control:GFP construct carries a fusion between the 5'-UTR of *At5G66380* and *GFP*. The experiment was conducted as described in the legend of Figure 4.

Figure S7. Heatmap showing degradome evidence across degradome libraries

Nine libraries (columns; Table S2) were scanned for 77 Arabidopsis miRNA/mRNA alignments with bulges >=4 nucleotides and MFE ratios >= 0.8. Color codes indicate category of hits. Category 0: single highest peak on the transcript is at the putative slice site; category 1: peak at putative slice site is at a peak that is tied with one or more other sites for the highest value on the transcript; category 2: peak at putative slice site is less than the maximum but above the average for the transcript; category 3: data at putative slice site is less than the average for the transcript but > 1; category 4: one read at the putative slice site; gray bars: No data at putative slice site.

Figure S8. *BCBP* expression data

Data were obtained from the eFP browser (37).

Figure S9. Percentage of mismatches between plant miRNAs and their targets

The percentage of mismatches (y-axis) that occur at each position relative to the 5' end of the miRNA (x-axis) between conserved (blue) and non-conserved (red) *Arabidopsis* miRNAs and their 5'-RACE PCR validated cleavage targets. G:U pairings were treated as 0.5 base-paired. A total of 102 and 37 validated miRNA:mRNA duplexes were analyzed for conserved and non-conserved miRNAs, respectively. The frequencies of mispairs were normalized so that each miRNA family had equal weight. miRNAs were classified as conserved if found in Arabidopsis and at least one of the following species: rice, poplar, poppy, moss. List of targets is presented in Table S3.

Figure S10. Natural variation of the *BCBP* miR398 target site

(A) Sequences of the *BCBP* miR398 target site in a core collection of 48 Arabidopsis accessions. Accession numbers correspond to the ones of the resource center of INRA/Versailles (38). The cleavage site and the position of the bulge are indicated. The two polymorphic regions (indicated in red) were also the two unique ones found by aligning the accessions available from the 1001 genome project.

(B) Comparison of the miR398 target site of *BCBP*-like genes within several plant species. The *A. thaliana* sequence corresponds to the Col-0 sequence of At5G20230. Sequences can be accessed using the following numbers: *A. Lyrata*, D7LZZ2_ARALL; *B. napus*, ES983129; *B. oleracea*, AM394382; *R. raphanistrum*, RR1H304TF; *T. halophila*, E4MY68_THEHA; *B. Rapa*, Bra002283; *T. halophila*, Thhalv10014739m. Polymorphisms are indicated in red. The cleavage site is indicated by an arrow. The start codon is underlined.

SUPPLEMENTAL TABLE LEGENDS

Table S1. Putative miR398 targets identified by loosening the rules in a conventional target finder software

Potential miR398 targets were retrieved using the mirU software (39) with the following parameters: a score of 3.5, allowing six G:U pairs, one indel and five mismatches. Letters in red indicate mismatches with the miR398a sequence (in blue); letters in green G:U wobbles; the letter in pink indicates a bulge. Genes highlighted in yellow are targets of miR398 identified in previous works. The gene highlighted in blue was identified in this study.

Table S2. Arabidopsis degradome libraries analysed

Table S3. Predicted minimum free energies (MFE) of duplexes between miRNAs and targets that were experimentally validated by 5'-RACE PCR

This list of targets was also used in Figure S9.

Table S4. miRNA-target site alignments for sensors used in this study

Table S5. Primers used in this study

SUPPLEMENTAL REFERENCES

37. Winter, D., Vinegar, B., Nahal, H., Ammar, R., Wilson, G.V. and Provart, N.J. (2007) An "Electronic Fluorescent Pictograph" browser for exploring and analyzing large-scale biological data sets. *PLoS One*, **2**, e718.

38. McKhann, H.I., Camilleri, C., Bérard, A., Bataillon, T., David, J.L., Reboud, X., Le Corre, V., Caloustian, C., Gut, I.G. and Brunel, D. (2004) Nested core collections maximizing genetic diversity in Arabidopsis thaliana. *Plant J*, **38**, 193-202.

39. Zhang, Y. (2005) miRU: an automated plant miRNA target prediction server. *Nucleic acids research*, **33**, W701-704.

Figure S1

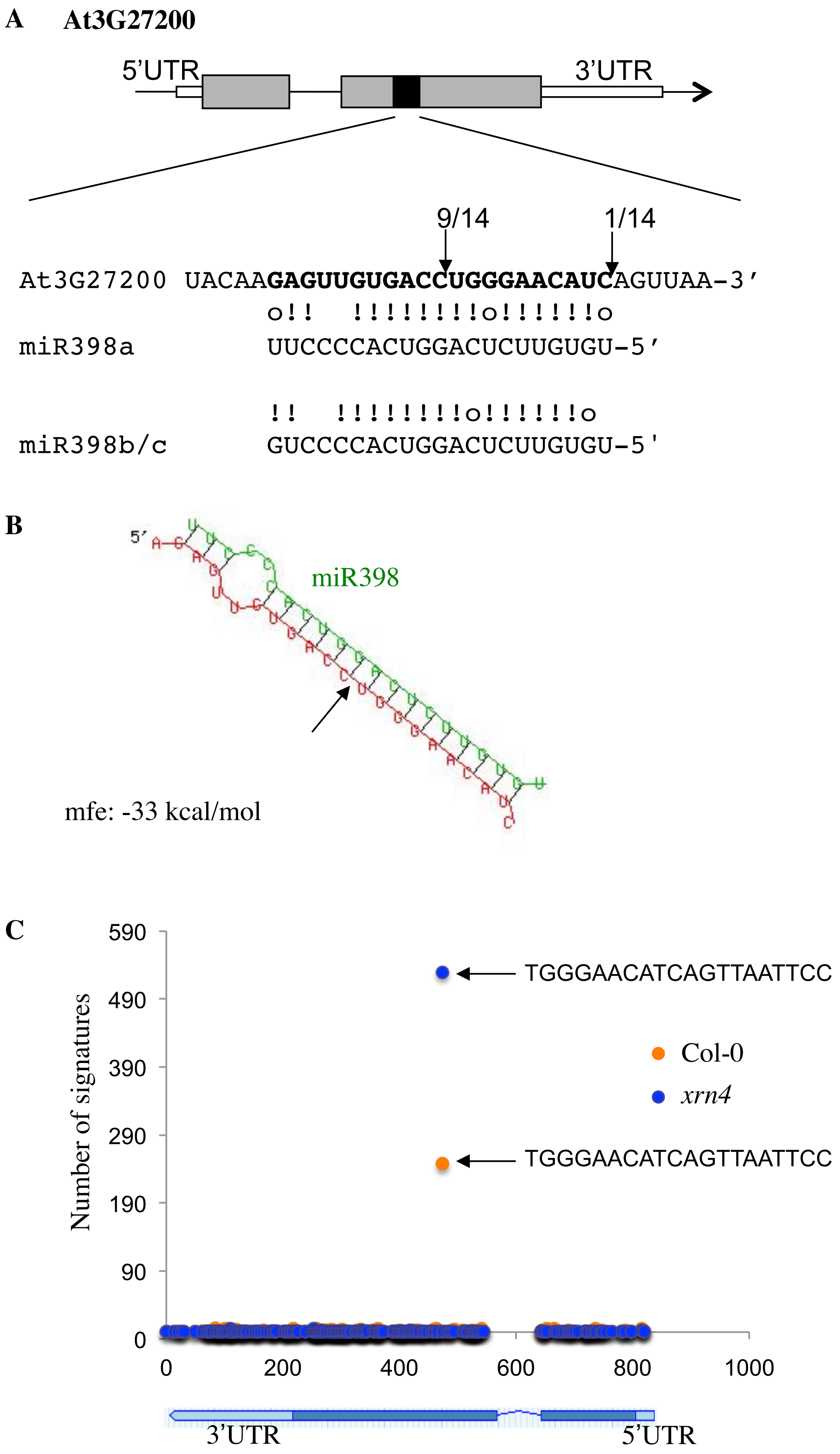
A
At3G27200
5'UTR
3'UTR
9/14
1/14
At3G27200 UACAAGAGUUGUGACCUGGGAACAUCAGUUAA-3'
o!! !!!!!!!!o!!!!!!o
miR398a UUCCCCACUGGACUCUUGUGU-5'
!! !!!!!!!!o!!!!!!o
miR398b/c GUCCCCACUGGACUCUUGUGU-5'
B
5'
miR398
mfe: -33 kcal/mol
C
Number of signatures
590
490
390
290
190
90
0
0
200
400
600
800
1000
TGGGAACATCAGTTAATTCC
Col-0
xrn4
TGGGAACATCAGTTAATTCC
3'UTR
5'UTR

Figure S2

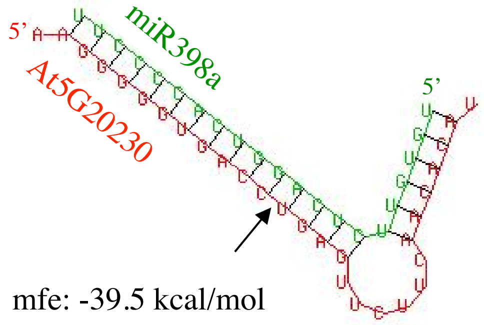

Figure S3

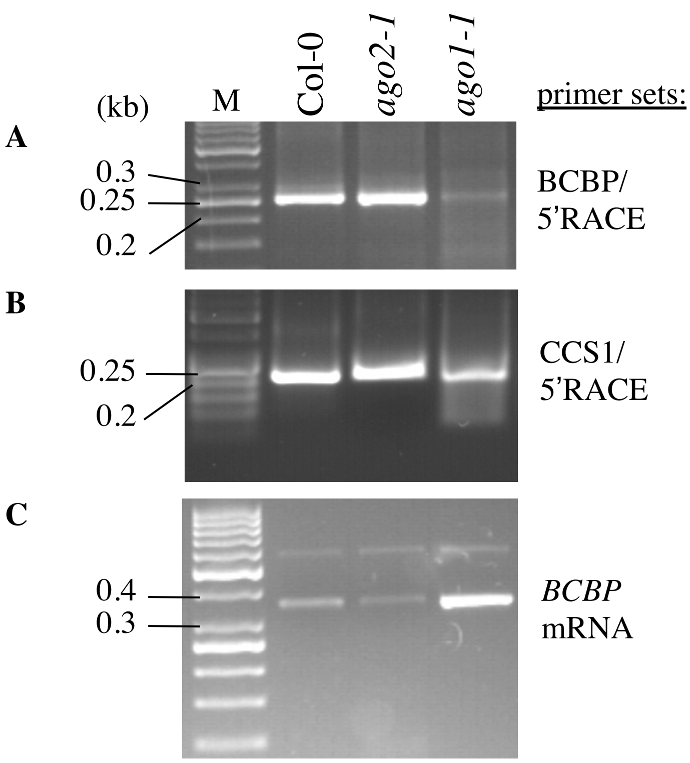
Col-0
ago2-1
ago1-1
(kb)
M
primer sets:
A
0.3
0.25
0.2
BCBP/
5'RACE
B
0.25
0.2
CCS1/
5'RACE
C
0.4
0.3
BCBP
mRNA

Figure S4

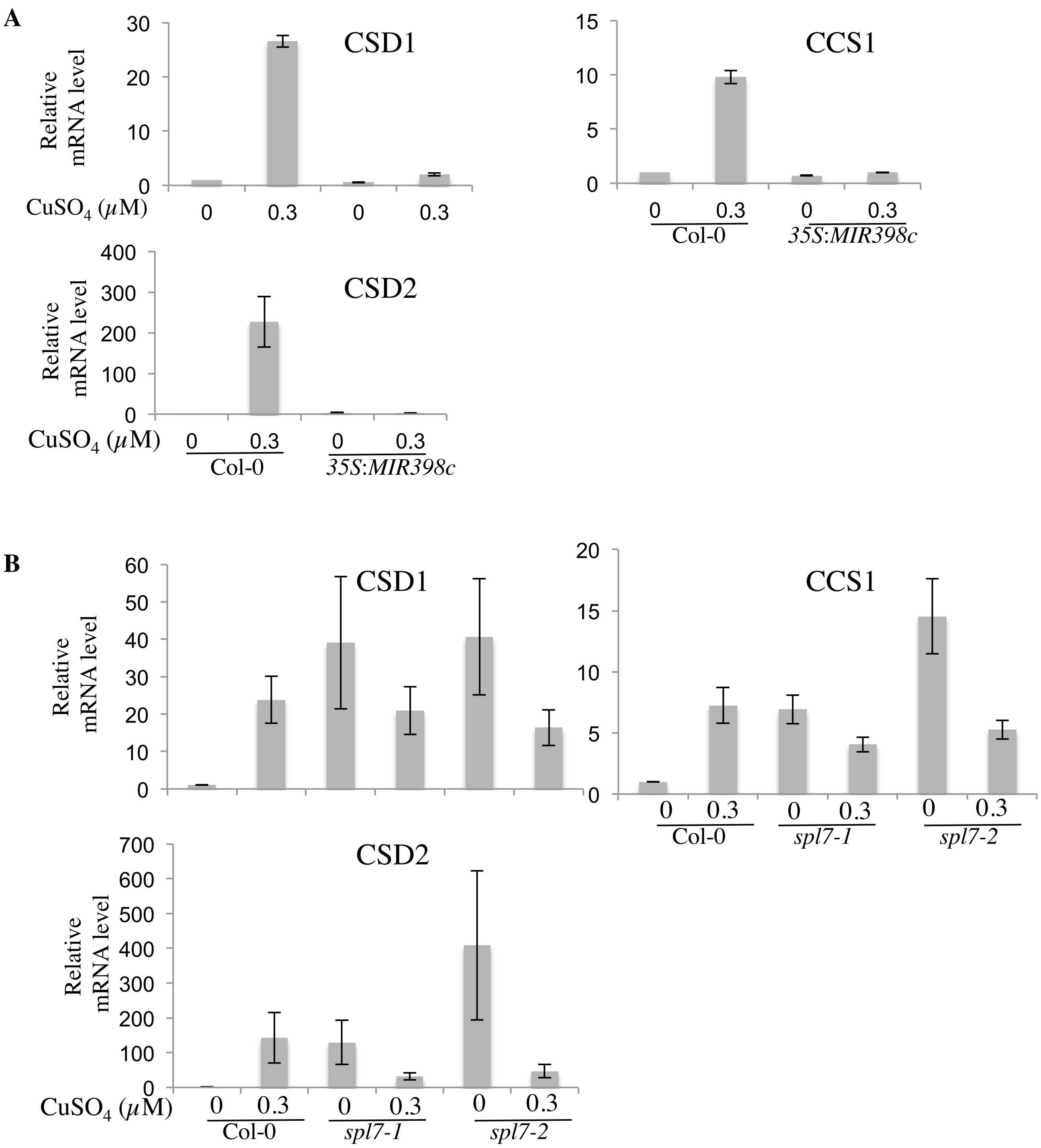

A
CSD1
Relative mRNA level
CuSO4 (μM)
0
0.3
0
0.3
CCS1
Col-0
35S:MIR398c
CSD2
B
CSD1
CCS1
spl7-1
spl7-2
CSD2

Figure S5

A

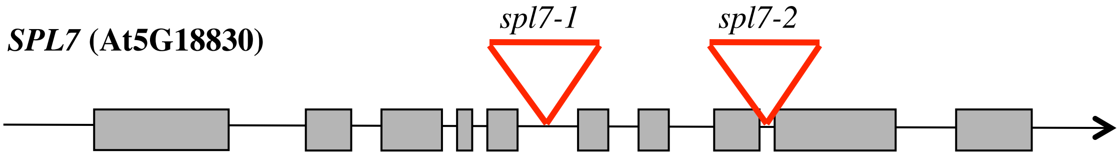

B

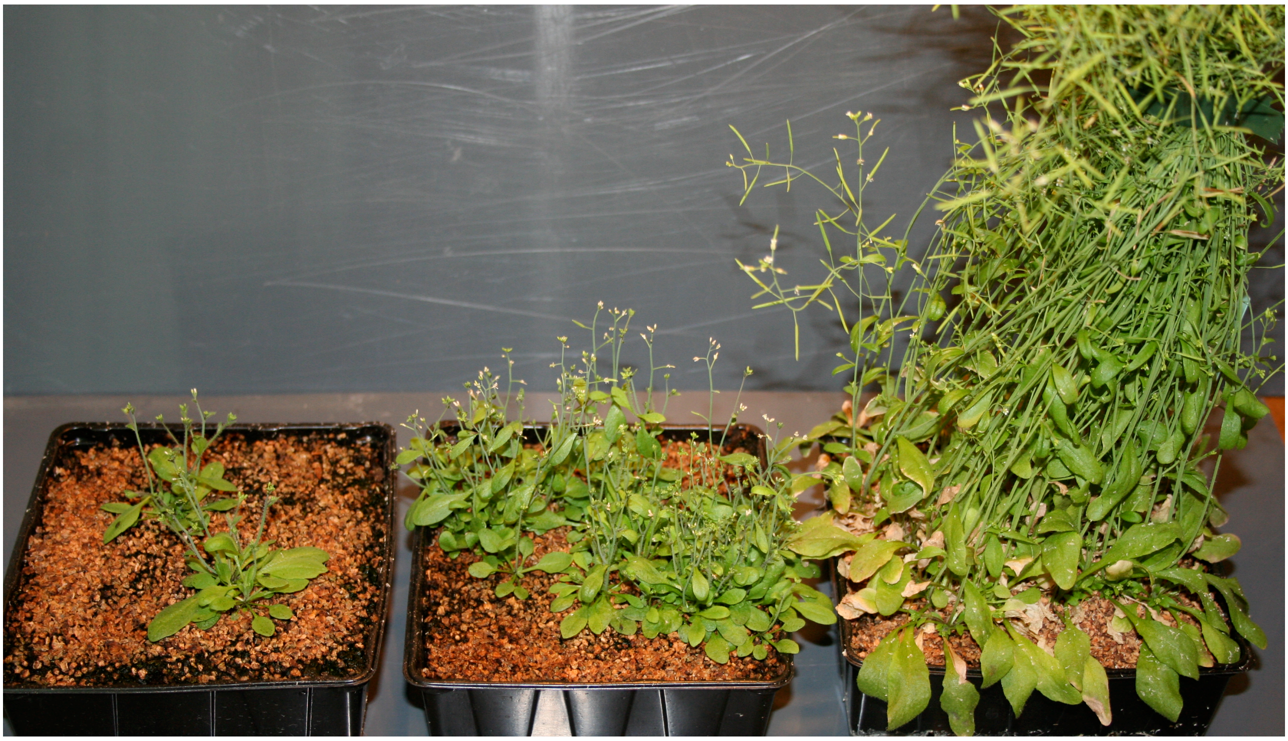

spl7-1 *spl7-2* Col-0

Figure S6

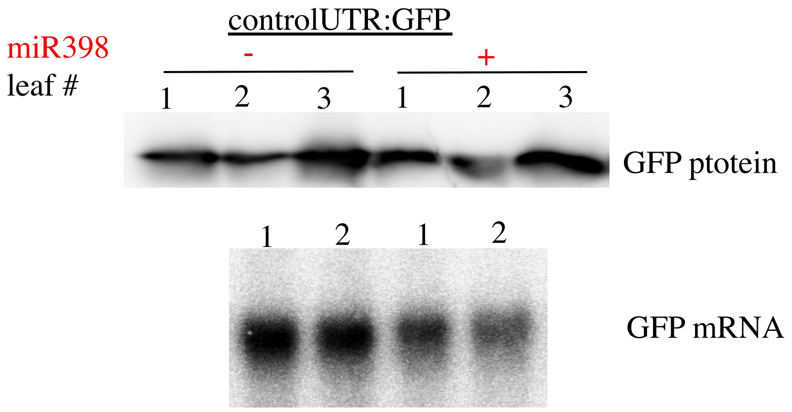
controlUTR:GFP
miR398
leaf #
-
+
1
2
3
1
2
3
GFP ptotein
1
2
1
2
GFP mRNA

Figure S7

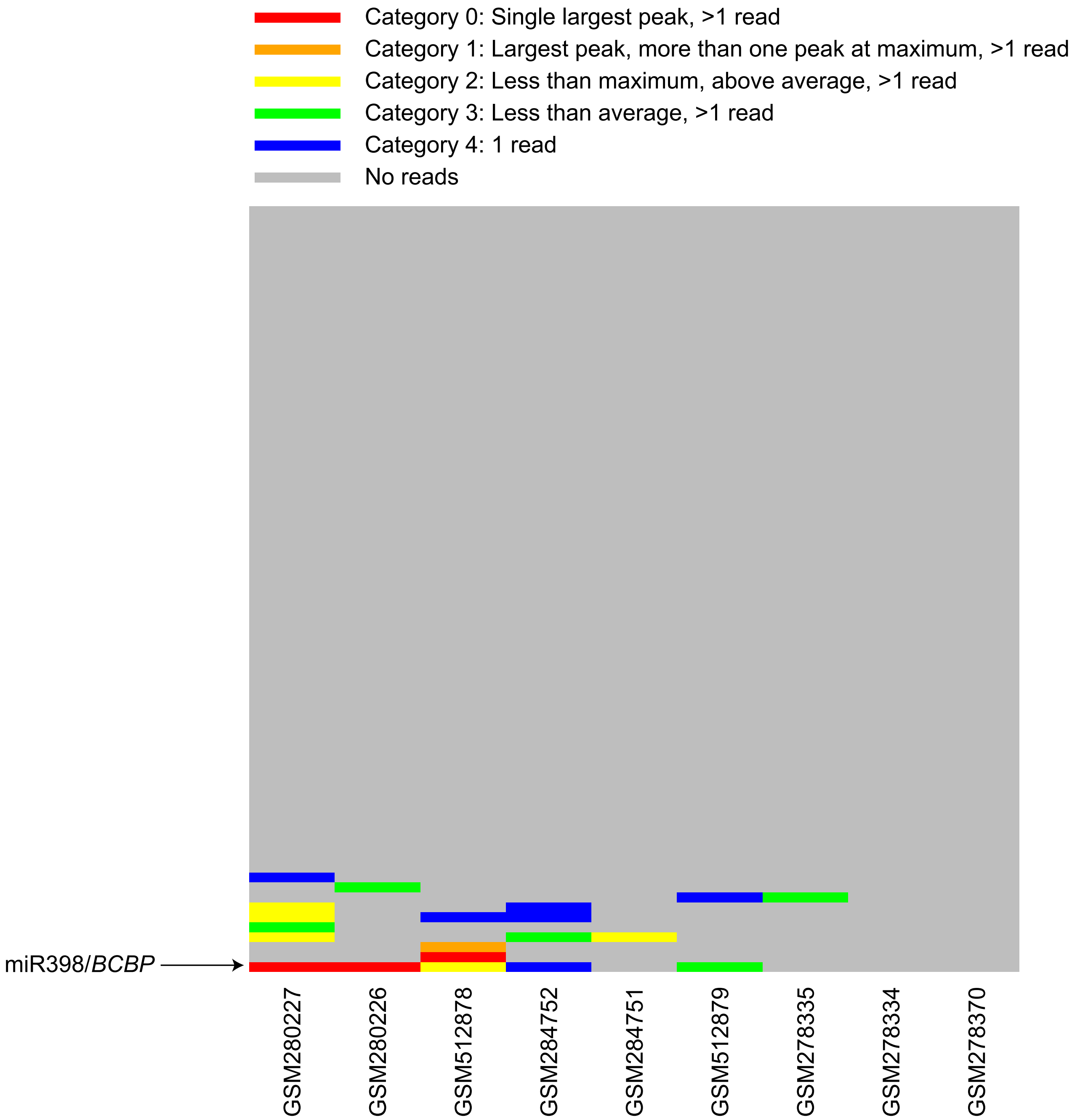

Category 0: Single largest peak, >1 read
Category 1: Largest peak, more than one peak at maximum, >1 read
Category 2: Less than maximum, above average, >1 read
Category 3: Less than average, >1 read
Category 4: 1 read
No reads
miR398/*BCBP*
GSM280227
GSM280226
GSM512878
GSM284752
GSM284751
GSM512879
GSM278335
GSM278334
GSM278370

Figure S8

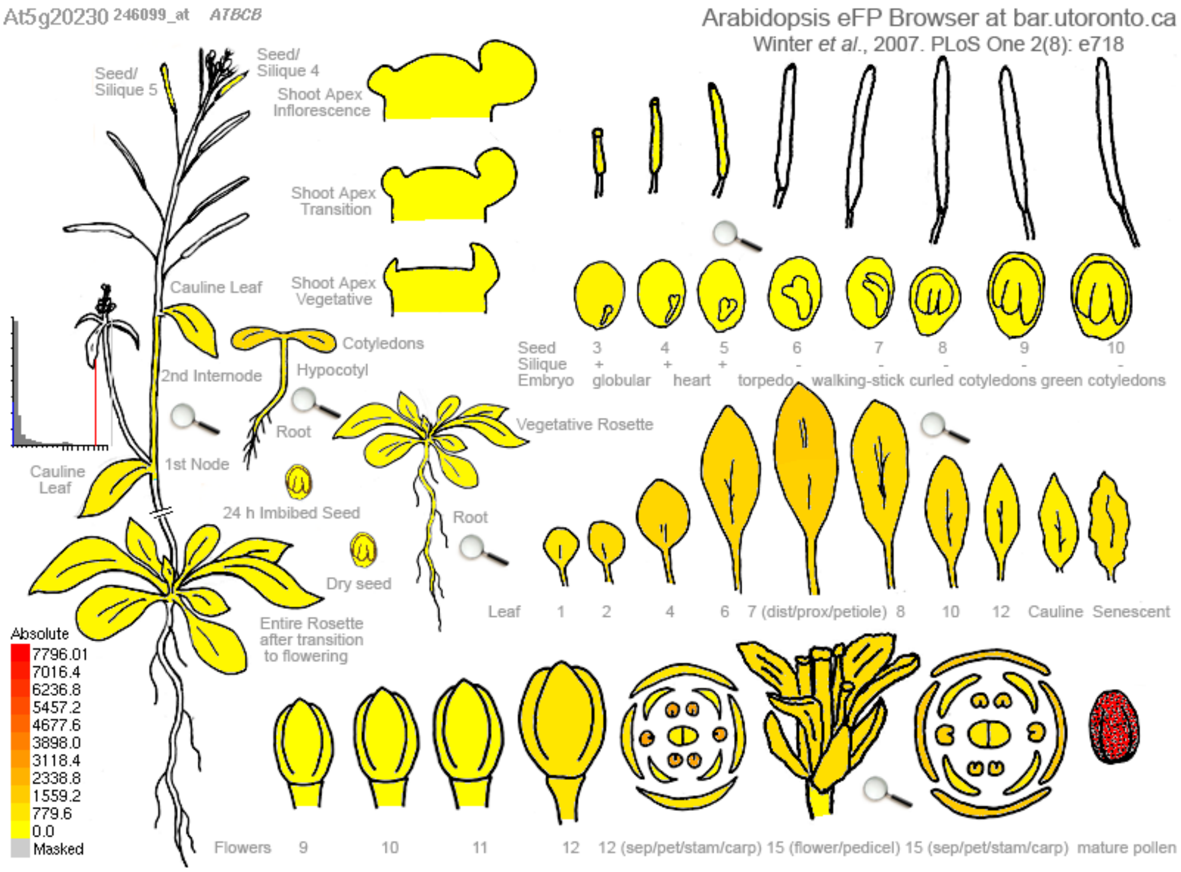
At5g20230 246099_at ATBCB
Arabidopsis eFP Browser at bar.utoronto.ca
Winter et al., 2007. PLoS One 2(8): e718
Seed/
Silique 5
Seed/
Silique 4
Shoot Apex
Inflorescence
Shoot Apex
Transition
Shoot Apex
Vegetative
Cauline Leaf
Cotyledons
Hypocotyl
2nd Internode
Root
1st Node
Cauline
Leaf
24 h Imbibed Seed
Vegetative Rosette
Root
Dry seed
Entire Rosette
after transition
to flowering
Seed 3 4 5 6 7 8 9 10
Silique + + + - - - - -
Embryo globular heart torpedo walking-stick curled cotyledons green cotyledons
Leaf 1 2 4 6 7 (dist/prox/petiole) 8 10 12 Cauline Senescent
Absolute
7796.01
7016.4
6236.8
5457.2
4677.6
3898.0
3118.4
2338.8
1559.2
779.6
0.0
Masked
Flowers 9 10 11 12 12 (sep/pet/stam/carp) 15 (flower/pedicel) 15 (sep/pet/stam/carp) mature pollen

Figure S9

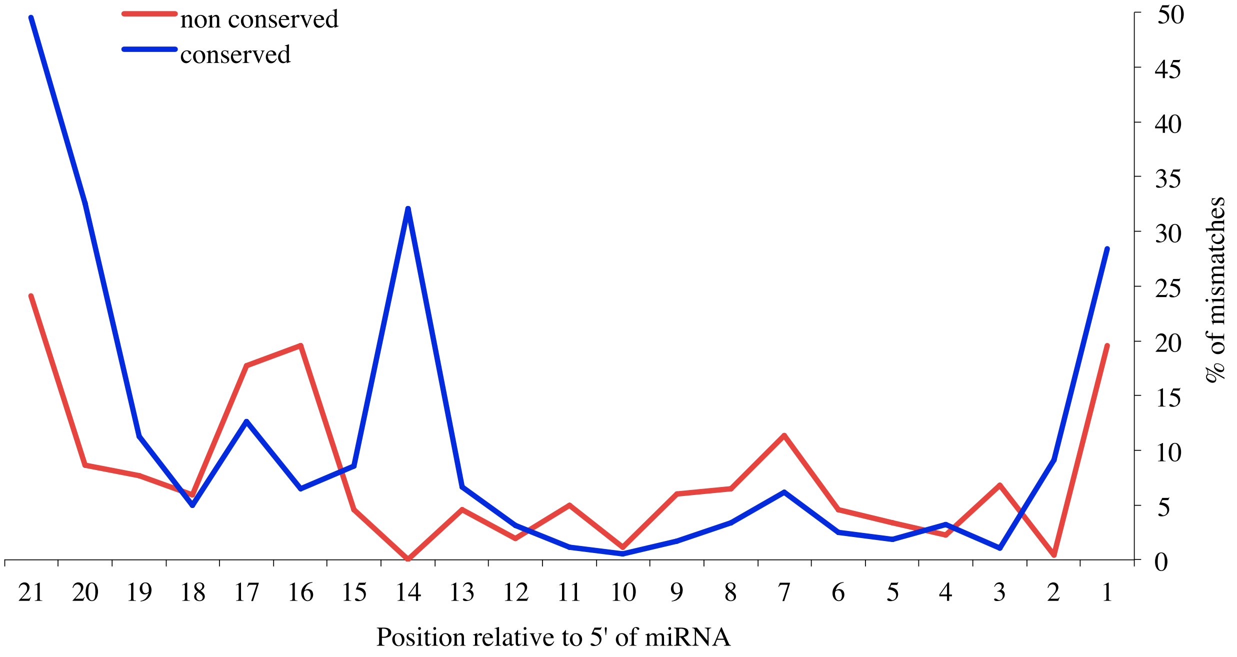

non conserved
conserved
50
45
40
35
30
25
20
15
10
5
0
% of mismatches
21 20 19 18 17 16 15 14 13 12 11 10 9 8 7 6 5 4 3 2 1
Position relative to 5' of miRNA

Figure S10

A

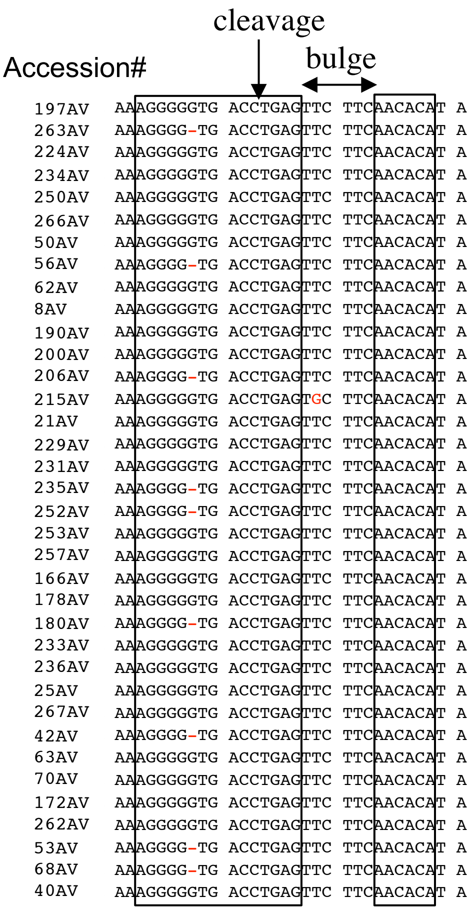

B

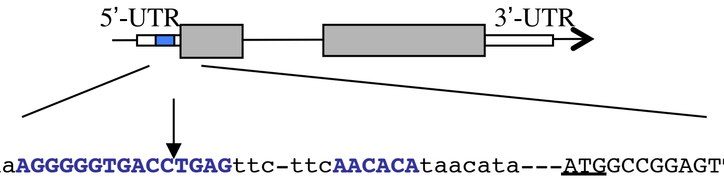


```
A. thaliana        aaaagaaaAGGGGGTGACCTGAGttc-ttcAACACAtaacata---ATGGCCGGAGTTTTCAAA
A. Lyrata          aaaagaaaaggggtgacctgagtgc-ttcaacacataacaat---ATGGCCGGAGTTTTCAAA
B. Napus/oleracea       aaaaggggtgacctgagttt-ttcaacacaacacaaag--ATGGCCGGAGTTTTCAAA
R. raphanistrum         aaaaggggtgacctgagtttttcaacacaacacataga-ATGGCCGGAGTTTTCAAA
R. sativus                  ggggtgacctgagttt-ttcaacacgacacaaa---ATGTCAGGATTATTCAAA
B. Rapa            aaaaaaaaa-ggggtgacctgagttt-ttcaacacaacacaaag--ATGGACGGAGTTTTCAAA
T. halophila                ggggtgacctgagttgtttcaacaca-aacacaaaaATGACCGGAGTTTTCAAA
```

Table S1. Putative miR398 targets identified by loosening the rules in a conventional target finder software

ID	Target Site Alignment	Site	Score	Mismatch
Query (3' - 5')	UUCCCCACUGGACUCUUGUGU			
At3g06370.1	uaggggugacuugaguacacc	2020 - 2040	3	4
At3g15640.1 cyt oxi (1)	aaggugugaccugagaa caca<br>u	95 - 116	3.5	2
At1g08830.1 CSD1 (1)	aaggggguuuccugagaucaca	92 - 112	3.5	3
At1g03630.1	caggguuguucagagaacaca	986 - 1006	3.5	5
At1g12520.1 CCS1 (2)	auugggagaccugggaacacu	691 - 711	3.5	5
At3g07760.1	aaaggaugaccagagaacauc	132 - 152	3.5	5
At4g26230.1	agguggugaccagagaguaca	102 - 122	3.5	5
At3g27200.1	gaguugugaccugggaacauc	256 - 276	3.5	6
At3g43860.1	ugggcgagaccugagaacaug	658 - 678	3.5	6
At4g11250.1	agacggugaucugagaacaug	132 - 152	3.5	6
At4g27510.1	aggggugacuuggaagcacu	1619 - 1639	3.5	6

References:

(1) Jones-Rhoades, M.W., and Bartel, D.P. (2004) Computational identification of plant microRNAs and their targets, including a stress-induced miRNA. *Mol Cell* 14, 787-799
(2) Beauclair L, Yu A, Bouché N. microRNA-directed cleavage and translational repression of the copper chaperone for superoxide dismutase mRNA in Arabidopsis. The Plant journal. 2010;62(3):454-62.

Table S2. Arabidopsis degradome libraries analysed

ID	Reference	Comments	Aligned Size	Distinct Positions
GSM278334	Addo-Quaye et al. (2008)	Infl, dT-primed, pool-amped	1 115 958	456 241
GSM278335	Addo-Quaye et al. (2008)	Infl, ran-primed, primer-extension	4 777 733	400 974
GSM278370	Addo-Quaye et al. (2008)	Seedlings, ran-primed, primer-extension	3 757 242	266 608
GSM280226	German et al. (2008)	Infl.	15 944 398	2 433 256
GSM280227	German et al. (2008)	Infl. - *xrn4*	8 680 036	3 533 771
AT1deg	Ma et al. (2010)	Infl. -- GSM512878 except from raw	8 207 435	666 623
AT2deg	Ma et al. (2010)	Infl. -- GSM512879 except from raw	8 917 199	857 704
GSM284751	Gregory et al. (2008)	immature floral	1 601 771	646 568
GSM284752	Gregory et al. (2008)	immature floral - *ein5-6*	2 892 581	1 077 362

References:

Addo-Quaye C, Eshoo TW, Bartel DP, Axtell MJ. Endogenous siRNA and miRNA targets identified by sequencing of the Arabidopsis degradome. Curr Biol. 2008;18(10):758-62.
German MA, Pillay M, Jeong DH, Hetawal A, Luo S, Janardhanan P, et al. Global identification of microRNA-target RNA pairs by parallel analysis of RNA ends. Nat Biotechnol. 2008;26(8):941-6.
Gregory BD, O'Malley RC, Lister R, Urich MA, Tonti-Filippini J, Chen H, et al. A link between RNA metabolism and silencing affecting Arabidopsis development. Dev Cell. 2008;14(6):854-66.
Ma Z, Coruh C, Axtell MJ. Arabidopsis lyrata small RNAs: transient MIRNA and small interfering RNA loci within the Arabidopsis genus. Plant Cell 2010 Apr;22(4):1090-103.

Table S3. Predicted minimum free energies (MFE) of duplexes between miRNAs and targets that were experimentally validated by 5'-RACE PCR

miRNA	AGI	Gene	MFE	Reference 5'-RACE PCR validation
miR156/157	AT2G33810	SPL3	-38.9	(3)
	AT1G27370	SPL10	-38.1	(4)
	AT1G53160	SPL4	-36.1	(3)
	AT5G43270	SPL2	-37.9	(5)
	AT3G15270	SPL5	-35.4	(3)
	AT2G42200	SPL9	-37.9	(17) (28)
	AT1G69170	SPL6	-38.2	(17) (18)
	AT1G27360	SPL11	-38.2	(17)
	AT5G50570	SPL13	-37.7	(17)
	AT3G57920	SPL15	-37.9	(17)
miR159	AT5G55930	OPT1	-32.3	(6)
	AT3G11440	MYB65	-37.7	(7) (4) (29)
	AT5G06100	MYB33	-37.7	(7) (29) (30)
	AT5G18100	CSD3	-26.6	(18)
	AT2G34010	MRG1	-33.1	(18) (20)
	AT2G32460	MYB101	-34.9	(20) (30)
	AT4G37770	ACS8	-32.1	(20)
	AT3G60460	MYB125	-35.4	(20)
	AT4G27330	NOZZLE1	-33.1	(21)
miR160	AT1G77850	ARF17	-51.4	(5) (8)
	AT2G28350	ARF10	-44.5	(5) (8)
	AT4G30080	ARF16	-44.9	(8)
miR162	AT1G01040	DCL1	-39.1	(9)
miR164	AT1G56010	NAC1	-43.4	(10)
	AT3G15170	CUC1	-44.9	(5) (10)
	AT5G07680	ANAC079/ANAC080	-44.3	(10)
	AT5G53950	CUC2	-44.6	(5) (10)
	AT5G61430	ANAC100	-44.2	(10)
	AT5G39610	NAC2	-37.9	(17)
miR165/166	AT1G30490	PHV	-42.3	(11)
	AT1G52150	ATHB-15	-42.8	(11)
	AT2G34710	PHB	-42.3	(11)
	AT4G32880	ATHB-8	-42.3	(11)
	AT5G60690	REV	-42.3	(12) (11)
miR167	AT5G37020	ARF8	-36.5	(5)
	AT1G51760	IAR3	-33.6	(22)
miR168	AT1G48410	AGO1	-38.5	(4)
	AT3G58030	zinc finger prot	-30.2	(18)
miR169	AT5G06510	NF-YA10	-37.5	(19)
	AT1G17590	NF-YA8	-39.1	unpublished data (N Bouché)
	AT1G72830	NF-YA3	-37.4	unpublished data (N Bouché)
	AT5G12840	AtHAP2A	-34.5	(18)
	AT1G54160	NF-YA5	-39.7	(23)
	AT3G05690	NF-YA2	-37.5	unpublished data (N Bouché)
miR170	AT3G60630	SCL6 III	-39.5	(13)
	AT4G00150	SCL6 IV	-39.8	(13)
	AT2G45160	SCL6 II	-39.5	(24)
miR172	AT2G28550	TOE1	-37.9	(5)
	AT4G36920	AP2	-38.8	(5)
	AT5G60120	TOE2	-39.5	(5)
	AT5G67180	TOE3	-37.7	(5)
	AT2g39250	SNZ	-35.7	(18) (31)
	AT3g54990	SMZ	-37.7	(18) (31)
	AT1G24793	ATLPXC1	nd	(25)
	AT5G16480	ATPFA-DSP5	nd	(25)
miR319	AT1G30210	TCP24	-39	(7)
	AT1G53230	TCP3	-39	(7)
	AT2G31070	TCP10	-39	(7)
	AT3G15030	TCP4	-39.4	(7)
	AT4G18390	TCP2	-39.1	(7)
miR390	AT3G17185	TAS3a	-38.2	(15)
miR393	AT1G12820	IPS1	-38.4	(14)
	AT3G23690	bHLH DNA-binding	-38.3	(14)
	AT3G26810	AFB2	-38.4	(14)
	AT3G62980	TIR1	-40.1	(14)
	AT4G03190	GRH1	-34.5	(14)
miR394	AT1G27340	F-box	-38.2	(14)
miR395	AT5G10180	SULTR2;1	-36.5	(15) (26)
	AT5G43780	APS4	-38.5	(14) (26)
	ATG322890	APS1	-34.4	(26)
miR396	AT2G36400	AtGRF3	-33.9	(14)
	AT4G24150	AtGRF8	-33.7	(14)
	AT5G53660	AtGRF7	-33.6	(14)
	AT2G22840	AtGRF1	-33.9	(14)
	AT4G37740	AtGRF2	-33.9	(14)
	AT2G45480	GRL9	-33.7	(14)
	AT1G10120	transc factor	nd	(17) (18)
	AT1G53910	RAP2.12	nd	(25)
	AT5G43060	MMG4.7	nd	(21)
	AT3G14110	FLU	nd	(21)
miR397	AT2G38080	IRX12 LAC4	-39.4	(14) (27)
	AT5G60020	LAC17	-37	(14) (27)
	AT2G29130	LAC2	-39.2	(14) (27)
	AT3G60250	PROTEIN KINASE CK2	-35	(18)
miR398	AT3G15640	cyt oxydase	-36	(14)
	AT2G28190	CSD2	-34.7	(14)
	AT1G08830	CSD1	-34.7	(14)
	AT1G12520	CCS1	-33.3	(19)
	AT3G27200	plastocyanin-like	-33	(25) this work
	AT5G20230	BCBP	-39.6	this work
miR399	AT2G33770	PHO2 site #2	-41	(15)
	AT2G33770	PHO2 site #3	-41.8	(15)
miR403	AT1G31280	AGO2	-38.4	(15)
miR408	AT2G30210	LAC3	-35.8	(6) (27)
	AT5G05390	LAC12	-37.9	(27)
	AT5G07130	LAC13	-37.9	(27)
	AT2G02850	plantacyanin	-45.5	(17) (18) (27)
	AT1G44790	Cu binding prot	nd	(18)
	AT1G72230	Cu binding prot	-36.5	(18)
	AT5G21930	PAA2	nd	(21)
	AT3G22110	PAC1	nd	(21)
miR472	AT1G51480	CC-NBS-LRR	-41.9	(16)
	AT5G43740	CC-NBS-LRR	-41.9	(16)

nd: not determined

<u>References:</u>

(1) Fahlgren, N., *et al*. (2007) High-throughput sequencing of Arabidopsis microRNAs: evidence for frequent birth and death of MIRNA genes. *PLoS ONE* 2, e219
(2) Jones-Rhoades, M.W., *et al*. (2006) MicroRNAS and their regulatory roles in plants. *Annu Rev Plant Biol* 57, 19-53
(3) Wu, G., and Poethig, R.S. (2006) Temporal regulation of shoot development in Arabidopsis thaliana by miR156 and its target SPL3. *Development* 133, 3539-3547
(4) Vazquez, F., *et al*. (2004) The nuclear dsRNA binding protein HYL1 is required for microRNA accumulation and plant development, but not posttranscriptional transgene silencing. *Curr Biol* 14, 346-351
(5) Kasschau, K.D., *et al*. (2003) P1/HC-Pro, a viral suppressor of RNA silencing, interferes with Arabidopsis development and miRNA unction. *Dev Cell* 4, 205-217
(6) Schwab, R., *et al*. (2005) Specific effects of microRNAs on the plant transcriptome. *Dev Cell* 8, 517-527

(7) Palatnik, J.F., *et al*. (2003) Control of leaf morphogenesis by microRNAs. *Nature* 425, 257-263
(8) Mallory, A.C., *et al*. (2005) MicroRNA-Directed Regulation of Arabidopsis AUXIN RESPONSE FACTOR17 Is Essential for Proper Development and Modulates Expression of Early Auxin Response Genes. *Plant Cell* 17, 1360-1375
(9) Xie, Z., *et al*. (2003) Negative feedback regulation of Dicer-Like1 in Arabidopsis by microRNA-guided mRNA degradation. *Curr Biol* 13, 784-789
(10) Mallory, A.C., *et al*. (2004) MicroRNA regulation of NAC-domain targets is required for proper formation and separation of adjacent embryonic, vegetative, and floral organs. *Current biology* 14, 1035-1046
(11) Mallory, A.C., *et al*. (2004) MicroRNA control of PHABULOSA in leaf development: importance of pairing to the microRNA 5' region. *Embo J* 23, 3356-3364
(12) Emery, J.F., *et al*. (2003) Radial patterning of Arabidopsis shoots by class III HD-ZIP and KANADI genes. *Curr Biol* 13, 1768-1774
(13) Llave, C., *et al*. (2002) Cleavage of Scarecrow-like mRNA targets directed by a class of Arabidopsis miRNA. *Science* 297, 2053-2056
(14) Jones-Rhoades, M.W., and Bartel, D.P. (2004) Computational identification of plant microRNAs and their targets, including a stress-induced miRNA. *Mol Cell* 14, 787-799
(15) Allen, E., *et al*. (2005) microRNA-directed phasing during trans-acting siRNA biogenesis in plants. *Cell* 121, 207-221
(16) Lu, C., *et al*. (2006) MicroRNAs and other small RNAs enriched in the Arabidopsis RNA-dependent RNA polymerase-2 mutant. *Genome Res* 16, 1276-1288
(17) Addo-Quaye C, Eshoo TW, Bartel DP, Axtell MJ. Endogenous siRNA and miRNA targets identified by sequencing of the Arabidopsis degradome. Curr Biol. 2008;18(10):758-62
(18) German MA, Pillay M, Jeong DH, Hetawal A, Luo S, Janardhanan P, et al. Global identification of microRNA-target RNA pairs by parallel analysis of RNA ends. Nat Biotechnol. 2008;26(8):941-6.
(19) Beauclair L, Yu A, Bouché N. microRNA-directed cleavage and translational repression of the copper chaperone for superoxide dismutase mRNA in Arabidopsis. The Plant journal. 2010;62(3):454-62.
(20) Alves-Junior L, Niemeier S, Hauenschild A, Rehmsmeier M, Merkle T. Comprehensive prediction of novel microRNA targets in Arabidopsis thaliana. Nucleic Acids Res. 2009;37(12):4010-21.
(21) Chorostecki U, Crosa VA, Lodeyro AF, Bologna NG, Martin AP, Carrillo N, et al. Identification of new microRNA-regulated genes by conserved targeting in plant species. Nucleic Acids Res. 2012;40(18):8893-904.
(22) Kinoshita N, Wang H, Kasahara H, Liu J, Macpherson C, Machida Y, et al. IAA-Ala Resistant3, an evolutionarily conserved target of miR167, mediates Arabidopsis root architecture changes during high osmotic stress. Plant Cell. 2012;24(9):3590-602.
(23) Li WX, Oono Y, Zhu J, He XJ, Wu JM, Iida K, et al. The Arabidopsis NFYA5 transcription factor is regulated transcriptionally and posttranscriptionally to promote drought resistance. Plant Cell. 2008;20(8):2238-51.
(24) Wang L, Mai YX, Zhang YC, Luo Q, Yang HQ. MicroRNA171c-targeted SCL6-II, SCL6-III, and SCL6-IV genes regulate shoot branching in Arabidopsis. Molecular plant. 2010;3(5):794-806.
(25) Zheng Y, Li YF, Sunkar R, Zhang W. SeqTar: an effective method for identifying microRNA guided cleavage sites from degradome of polyadenylated transcripts in plants. Nucleic Acids Res. 2012;40(4):e28.
(26) Kawashima CG, Yoshimoto N, Maruyama-Nakashita A, Tsuchiya YN, Saito K, Takahashi H, et al. Sulphur starvation induces the expression of microRNA-395 and one of its target genes but in different cell types. Plant J. 2009;57(2):313-21.
(27) Abdel-Ghany SE, Pilon M. MicroRNA-mediated systemic down-regulation of copper protein expression in response to low copper availability in Arabidopsis. J Biol Chem. 2008;283(23):15932-45.
(28) Wang JW, Schwab R, Czech B, Mica E, Weigel D. Dual effects of miR156-targeted SPL genes and CYP78A5/KLUH on plastochron length and organ size in Arabidopsis thaliana. Plant Cell. 2008;20(5):1231-43.
(29) Allen RS, Li J, Stahle MI, Dubroue A, Gubler F, Millar AA. Genetic analysis reveals functional redundancy and the major target genes of the Arabidopsis miR159 family. Proc Natl Acad Sci U S A. 2007;104(41):16371-6.
(30) Reyes JL, Chua NH. ABA induction of miR159 controls transcript levels of two MYB factors during Arabidopsis seed germination. Plant J. 2007;49(4):592-606.
(31) Mathieu J, Yant LJ, Murdter F, Kuttner F, Schmid M. Repression of flowering by the miR172 target SMZ. PLoS Biol. 2009;7(7):e1000148.

Table S4. miRNA-target site alignments for sensors used in this study

<table>
<tr><th>Target</th><th>Sequence and Complementarity</th></tr>
<tr><td>Spacer</td><td>5'-AUCGGUAUCCAGCUAGUACAA-3' Spacer
   ||*|*****o****|
3'-GUCCCCACUGGACUCUUGUGU-5' miR398b</td></tr>
<tr><td>Perfect</td><td>5'-CAGGGGUGACCUGAGAACACA-3' Perfect
   |||||||||||||||||||||
3'-GUCCCCACUGGACUCUUGUGU-5' miR398m</td></tr>
<tr><td>*BCBP*</td><td>5'-AGGGGGUGACCUGAGUUCUUCAACACA-3' *BCB_m*
   *o||||||||||||| ||||||
3'-GUCCCCACUGGACUC------UUGUGU-5' miR398b</td></tr>
<tr><td>Bulge_3</td><td>5'-CAGGGGUGACCUGAGAACAUUCUUCCA-3' Bulge_3
   |||||||||||||||||||o*
3'-GUCCCCACUGGACUCUUGUGU-5' miR398b</td></tr>
<tr><td>Bulge_4</td><td>5'-CAGGGGUGACCUGAGAACUUCUUGACA-3' Bulge_4
   ||||||||||||||||||***
3'-GUCCCCACUGGACUCUUGUGU-5' miR398b</td></tr>
<tr><td>Bulge_5</td><td>5'-CAGGGGUGACCUGAGAAUUCUUCCACA-3' Bulge_5
   |||||||||||||||||o***
3'-GUCCCCACUGGACUCUUGUGU-5' miR398b</td></tr>
<tr><td>Bulge_6</td><td>5'-CAGGGGUGACCUGAGAUUCUUCACACA-3' Bulge_6
   |||||||||||||||| |||||
3'-GUCCCCACUGGACUCU------UGUGU-5' miR398b</td></tr>
<tr><td>Bulge_7</td><td>5'-CAGGGGUGACCUGAGUUCUUCAACACA-3' Bulge_7
   ||||||||||||||| ||||||
3'-GUCCCCACUGGACUC------UUGUGU-5' miR398b</td></tr>
<tr><td>Bulge_8</td><td>5'-CAGGGGUGACCUGAUUCUUCGAACACA-3' Bulge_8
   |||||||||||||| |||||||
3'-GUCCCCACUGGACU------CUUGUGU-5' miR398b</td></tr>
<tr><td>Bulge_9</td><td>5'-CAGGGGUGACCUGUUCUUCAGAACACA-3' Bulge_9
   ||||||||||||| ||||||||
3'-GUCCCCACUGGAC------UCUUGUGU-5' miR398b</td></tr>
<tr><td>Bulge_10</td><td>5'-CAGGGGUGACCUUUCUUCGAGAACACA-3' Bulge_10
   |||||||||||| |||||||||
3'-GUCCCCACUGGA------CUCUUGUGU-5' miR398b</td></tr>
<tr><td>Bulge_11</td><td>5'-CAGGGGUGACCAUCUUGUGAGAACACA-3' Bulge_11
   ||||||||||| ||||||||||
3'-GUCCCCACUGG------ACUCUUGUGU-5' miR398b</td></tr>
<tr><td>Bulge_12</td><td>5'-CAGGGGUGACUUCUUGCUGAGAACACA-3' Bulge_12
   |||||||||| |||||||||||
3'-GUCCCCACUG------GACUCUUGUGU-5' miR398b</td></tr>
<tr><td>Bulge_13</td><td>5'-CAGGGGUGAUUCUUCCCUGAGAACACA-3' Bulge_13
   ||||||||| ||||||||||||
3'-GUCCCCACU------GGACUCUUGUGU-5' miR398b</td></tr>
<tr><td>Bulge_14</td><td>5'-CAGGGGUGUUCUUCACCUGAGAACACA-3' Bulge_14
   |||||||| |||||||||||||
3'-GUCCCCAC------UGGACUCUUGUGU-5' miR398b</td></tr>
<tr><td>Bulge_15</td><td>5'-CAGGGGUUUCUUCGACCUGAGAACACA-3' Bulge_15
   ||||||| ||||||||||||||
3'-GUCCCCA------CUGGACUCUUGUGU-5' miR398b</td></tr>
<tr><td>Bulge_16</td><td>5'-CAGGGGAUCUUCUGACCUGAGAACACA-3' Bulge_16
   |||||| |||||||||||||||
3'-GUCCCC------ACUGGACUCUUGUGU-5' miR398b</td></tr>
<tr><td>Bulge_17</td><td>5'-CAGGGUUCUUCGUGACCUGAGAACACA-3' Bulge_17
   ||||| ||||||||||||||||
3'-GUCCC------CACUGGACUCUUGUGU-5' miR398b</td></tr>
<tr><td>Bulge_18</td><td>5'-CAGGUUCUUCGGUGACCUGAGAACACA-3' Bulge_18
   |||| |||||||||||||||||
3'-GUCC------CCACUGGACUCUUGUGU-5' miR398b</td></tr>
<tr><td>Bulge_19</td><td>5'-CAGUUCUUCGGGUGACCUGAGAACACA-3' Bulge_19
         ***||||||||||||||||||
      3'-GUCCCCACUGGACUCUUGUGU-5' miR398b</td></tr>
<tr><td>Bulge_20</td><td>5'-CAUUCUUCGGGGUGACCUGAGAACACA-3' Bulge_20
         **|||||||||||||||||||
      3'-GUCCCCACUGGACUCUUGUGU-5' miR398b</td></tr>
</table>

Table S5. Primers used in this study

qPCR expression analyses

gene	oligo set	5'-3' sequence
BCBP	At5G20230f_qPCR	TAGTTCTCGACCAAGAAAAG
	At5G20230r_qPCR	CCATTCCGTATCATCACC
CSD1	CSD1F4	GATAGCTCAAGCACTTGATTCTTTCC
	CSD1R4	GTCACACCATCGCCTTCCTGGGTG
CSD2	CSD2F2	CAGAAGATGAGTGCCGTCATGCGG
	CSD2R2	CCGAGGTCATCCTTAAGCTCGTG
CCS1	CCS1F	CCCATATGACAGTACCATCA
	CCS1R	CCATTTCAAGATCAAACTGGCAC

5'RACE PCR

gene	Oligo usage	oligo set nested PCR#1*	5'-3' sequence	oligo set nested PCR#2*	5'-3' sequence
BCBP	5'RACE	20230_n	GCAGCTTCTGATACAACTG	20230_n2	CATCATGCCTCCCAGCAGC
		5'RACE	GGACACTGACATGGACTGAAGGAGTA	5'RACE	GGACACTGACATGGACTGAAGGAGTA
GFP	5'RACE	sGFP_R2	GTGCAGATGAACTTCAGGGTC	sGFP_R	GTAGGTGGCATCGCCCTCGCC
		5'RACE	GGACACTGACATGGACTGAAGGAGTA	5'RACE	GGACACTGACATGGACTGAAGGAGTA
CCS1	5'RACE	CCS1_n2	GTAGTTTTCTCCAACCCCTG	CCS1_n	CCCATATGACAGTACCATCA
		5'RACE	GGACACTGACATGGACTGAAGGAGTA	5'RACE	GGACACTGACATGGACTGAAGGAGTA
BCBP	mRNA amplification	At5G20230 F	GGGGTCATTTACTATCTTT	-	-
		At5G20230 R	GTTTCTCTTTCTCACAGTTTTC	-	-

*nested PCRs were done by using first PCR#1 and then PCR#2 oligo sets

Design of Constructs

construct	vectors	oligo set	5'-3' sequence
proBCBP:BCBP	pDONR207/pGWB1	At5G20230 attB1 F	GGGGACAAGTTTGTACAAAAAAGCAGGCTCGGGTTATGACCGCATTCG
		At5G20230 attB2 R3	GGGGACCACTTTGTACAAGAAAGCTGGGTGAATTCAGGTGAGAAACGTATCCAC
proBCBP:2mBCBP	pDONR207/pGWB1	At5g20230 mut F2	GAAAAGAAAAGGGGGTGACGTCAGTTCTTCAACACATAACATA
		At5g20230 mut R2	TATGTTATGTGTTGAAGAACTGACGTCACCCCCTTTTCTTTTC
BCBP UTRs:GFP	pDONR207/pGWB5	attB1 20230 5UTR F	GGGGACAAGTTTGTACAAAAAAGCAGGCTGAAACCACAACAAACACAAAAATC
		attB2 20230 5UTR R	GGGGACCACTTTGTACAAGAAAGCTGGGTACATTATGTTATGTGTTGAAGAACTC
controlUTR:GFP	pDONR207/pGWB5	attB1 AT5G66380 F	GGGGACAAGTTTGTACAAAAAAGCAGGCTCTTGAACGGACGGCAGAAC
		attB1 AT5G66380 R	GGGGACCACTTTGTACAAGAAAGCTGGGTACATCAACGAATCCTTAGATG

Probes

Probe	oligo set	5'-3' sequence
GFP/northern	GFP F	CAAGGACGACGGCAACTAC
	GFP R	GCTTGTCGGCCATGATATAG

Luciferase experiments

Name	Purpose	5'-3' sequence
35SF	Amplify 35S promoter	GGGCCCACCCCCCTACTCCAAAAATG
35SR	Amplify 35S promoter	CTCGAGGTCCTCTCCAAATGAAATG
TerF	Amplify 35S terminator	TCCACTAGTTAATGTGTGAGTAGTTTCCCG
TerR	Amplify 35S terminator	GTGGCGGCCGCGATCTGGATTTTAGTACTGGA
UTRs	Hybridize with UTRas to	GTCGCCATGCGGTCATTACACTATCCCTAGGA
UTRas	Hybridize with UTRs to	CTAGTTCAAGAGTAAAAGATAGTAAAACCGG
RlucF	Amplify *Renilla* luciferase	GTCGACATGACTTCGAAAGTTTATGATCC
RlucR	Amplify *Renilla* luciferase	TCTAGATTATTGTTCATTTTTGAGAACTCGC
FlucF	Amplify firefly luciferase	CTCGAGATGGAAGACGCCAAAAACATAAAGA
FlucR1	Amplify firefly luciferase	GAATTCTTACACGGCGATCTTTCCGCCCTTC
FlucR2	Amplify firefly	GAATTCCACGGCGATCTTTCCGCCCTTC
Rluc_intF	Real-time PCR for Rluc	TGTTGGACGACGAACTTCAC
Rluc_intR	Real-time PCR for Rluc	CATTTTTGTCGGCCATGATT
Fluc_clvF	Real-time PCR for Fluc	GTTTTGGAGCACGGAAAGAC
Fluc_clvR	Real-time PCR for Fluc	CAAGAGTAAAAGATAGTAAAACCGG
